# A multi-objective supplier selection framework based on user-preferences

**DOI:** 10.1007/s10479-021-04251-5

**Published:** 2021-10-21

**Authors:** Federico Toffano, Michele Garraffa, Yiqing Lin, Steven Prestwich, Helmut Simonis, Nic Wilson

**Affiliations:** 1 Insight Centre for Data Analytics, School of Computer Science and IT, University College Cork, Cork, Ireland; 2 United Technologies Research Centre, Cork, Ireland; 3 School of Computer Science and IT, University College Cork, Cork, Ireland; 4 United Technologies Research Centre, East Hartford, USA

**Keywords:** Supplier selection, Preference elicitation, Incremental elicitation, Multi-attribute utility theory, Multi-objective optimization, Mathematical programming

## Abstract

This paper introduces an interactive framework to guide decision-makers in a multi-criteria supplier selection process. State-of-the-art multi-criteria methods for supplier selection elicit the decision-maker’s preferences among the criteria by processing pre-collected data from different stakeholders. We propose a different approach where the preferences are elicited through an active learning loop. At each step, the framework optimally solves a combinatorial problem multiple times with different weights assigned to the objectives. Afterwards, a pair of solutions among those computed is selected using a particular query selection strategy, and the decision-maker expresses a preference between them. These two steps are repeated until a specific stopping criterion is satisfied. We also introduce two novel fast query selection strategies, and we compare them with a myopically optimal query selection strategy. Computational experiments on a large set of randomly generated instances are used to examine the performance of our query selection strategies, showing a better computation time and similar performance in terms of the number of queries taken to achieve convergence. Our experimental results also show the usability of the framework for real-world problems with respect to the execution time and the number of loops needed to achieve convergence.

## Introduction

Supplier selection is the process of determining the best suppliers for acquiring the necessary materials for the production activities of a firm. This is a key aspect of Operations Management (OM) for a firm of any size. Although decision-makers (DMs) still proceed manually in some contexts, many automated methods and tools have been adopted to solve the problem. The main benefits of using these instruments include the reduction of the decision process time and the capability to take into account complex aspects arising when the business grows. These frameworks do not merely select the least expensive suppliers. They can also consider multiple criteria (such as lead time, product quality, resilience, suppliers’ reputation and relationship, etc.) to sharpen the company’s competitiveness. The task of quantifying the relative importance of such criteria in a specific decision process can be tricky, partly because it involves a variety of factors and business goals. This is usually achieved by conducting long interviews with multiple experts and stakeholders.

Multi-criteria supplier selection problems have been widely studied in the last few decades. Numerous operations research approaches have been developed to address the different challenges. A common way to handle multiple criteria is to evaluate the different alternatives through a utility function defined as the weighted sum of the criteria considered. In this context, the problem can be decomposed into two major tasks:

- Determining the weights by eliciting the DM’s preferences on the criteria;
- Solving the problem given some fixed weights of the criteria.

Recent works on multi-criteria supplier selection follow this general structure. This eases the development of hybrid approaches based on a pair of techniques, one for each of the two tasks. The first task is generally covered by Multicriteria Decision Making (MCDM) (or multi-criteria decision analysis) methods, such as the Analytic Hierarchy Process (AHP), the Analytic Network Process (ANP) or fuzzy-based extensions taking into account incomplete data (Ortiz Barrios et al. 2020; Chang 2019; Bodaghi et al. 2018; Ecer 2020; Shaw et al. 2012). These methods are based on structural tables with elements of ambiguous stakeholder opinions which can be synthesized to define the weights of the criteria. Alternatively, the weights may be considered as given constants (Suprasongsin et al. 2019), or they may be converted into a profit or cost measure (Ventura et al. 2020; Arampantzi et al. 2019; Andrade-Pineda et al. 2017). The second task consisted of ranking a set of alternative suppliers or choosing a certain supplier configuration. The latter involves solving a combinatorial optimization problem, usually employing Mathematical Programming (MP) (Bodaghi et al. 2018; Ortiz Barrios et al. 2020; Kaur and Singh 2019), or metaheuristic techniques (Hashim et al. 2017; Rezaei and Davoodi 2011).

**Fig. 1:**
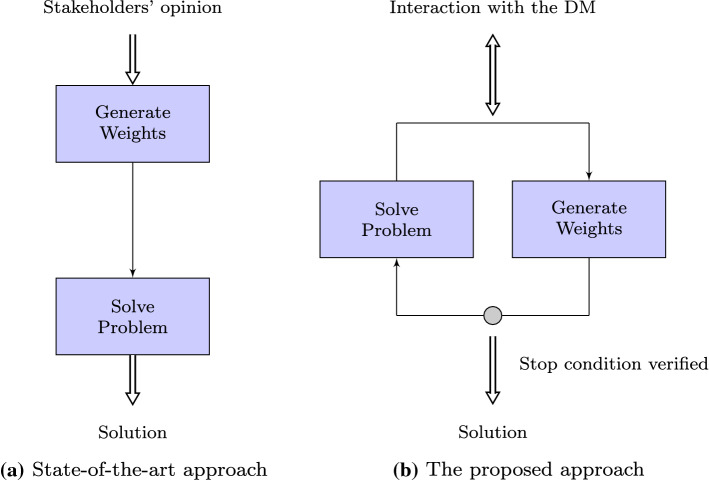
Comparison of the decompositions used to solve multi-criteria supplier selection problems

Our first research question is the following. How can we reduce the cognitive effort required to learn the criteria weights of a multi-objective supplier selection problem? We addressed this question by introducing an active learning approach to the supplier selection problem. Active learning is an Artificial Intelligence (AI) technique where the learning algorithm is allowed to choose the data from which it learns (Settles (2012)). We adopted this technique for an interactive preference elicitation process in order to iteratively reduce the uncertainty of the DM’s preferences (see, e.g., Korhonen 2005; Benabbou et al. 2020). To the best of our knowledge, this approach has never been used before for supplier selection. Figure 1 shows the fundamental difference between the standard methods (Fig. 1a) and an active learning approach (Fig. 1b). Briefly, our framework asks the DM to provide a preference between two solutions at each iteration. The response is used to reduce the uncertainty regarding the weighted vector representing the DM’s preferences. This is a more straightforward method to elicit the DM’s preferences, when compared with standard techniques such as AHP that require a good understanding of the model itself to be set up properly (Whitaker 2007).

We also considered a second research question related to the query selection process of our active learning loop. Our framework evaluated the quality of a solution by considering the max regret of the utility function with respect to compatible preference models. A related myopically optimal query selection strategy in terms of the value of information is the setwise minimax regret criterion (Viappiani and Boutilier 2020). A key point for the usability of our framework is the formulation of questions for the DM with a high value of information since this can reduce the number of interactions with the DM. However, the setwise minimax regret criterion is expensive in terms of computational time, thus it can delay the interaction with the DM during the learning process. How can we reduce the query computation time while still generating high informative queries? We addressed this problem by proposing two very fast novel methods for query selection based on a measure that we call *discrepancy*.

The main contribution of this paper is therefore the development of an approach to a supplier selection problem based on interleaving elicitation and optimization, including two novel methods for generating queries for the decision-maker. This enables the preferred solutions to be found with the decision-maker having to answer only a fairly small number of natural queries involving pairwise comparisons between solutions.

This paper is organized as follows. Section 2 provides a literature review of the approaches developed for supplier selection (Sect. 2.1) and of general strategies for preference elicitation (Sect. 2.2). Our study was inspired by a real-world supplier selection problem with evaluation criteria, constraints and instance structure coming from a medium-size factory as part of a manufacturing corporation. The assumptions made in relation to the problem definition are discussed in Sect. 3. Some key mathematical notations used in the paper are presented in Sect. 4. The structure of the framework is described in Sect. 5. The two main blocks of the framework are:

- A Mixed Integer Linear Programming model used to solve the combinatorial optimization problem (described in Sect. 5.1);
- Preference Elicitation strategies for computing the queries posed to the user (described in Sect. 5.2).

Section 6 presents some of the computational results showing the performance of the framework. We conclude with Sect. 7 discussing the framework, including the implications for managers and decision-makers (Sect. 7.1), the implications for the theory (Sect. 7.2), and potential future works (Sect. 7.3).

## Literature review

This work fits within the scope of applying AI methods to improve decision making in modern factories. This is one of the pillars of the digital transformation brought about Industry 4.0. Recently, Grover et al. (2020) provided a survey with guidelines to managers on applying AI methods in different components of OM. AI methods aim at making decisions based on some knowledge that is extracted from a source of data. This has been performed successfully in many aspects of OM related to manufacturing, such as inventory optimization, the supply chain, planning and scheduling, product design, etc. In this work, we focused on a multi-criteria supplier selection, which is a fundamental aspect of OM (Verma and Pullman 1998; Choi and Hartley 1996; Chou and Chang 2008). One of the challenges of this task was determining the DM’s trade-offs among the evaluation criteria. One could consider historical data for this purpose. However, the firm’s strategy may change dynamically and depend on a number of intangible factors. We therefore adopted a preference learning technique based on iterative online queries, whose answers allowed us to reduce the uncertainty of the DM’s preferences. This approach is a technique that arose in the field of AI. Work on this topic appears in leading AI journals and at some of the most prestigious AI conferences (see, e.g., Chajewska et al. (2000); Boutilier (2002); Braziunas and Boutilier (2007, 2008); Viappiani and Boutilier (2020)). Our literature review focuses on the two main aspects of this paper. The first is supplier selection, which is a class of problems that are traditionally tackled using different MCDM, AI and optimization techniques. Section 2.1 reviews recent papers related to this topic by discussing the proposed methodologies and the different constraints and objectives included in the problem definition. The second aspect is related to AI methods used for preference elicitation, which is at the core of the approach proposed in this work. Section 2.2 provides an overview of previous work in this area.

### Approaches for supplier selection

The supplier selection literature is very rich with a wide variety of approaches that have been developed and tailored to solve specific versions of the problem, with different constraints/objectives. See, for instance, the surveys (Weber et al. 1991; Aissaoui et al. 2007; Ware et al. 2012; Zimmer et al. 2016) that provide a deep introduction to the quantitative and qualitative methods used. Recent advances in supplier selection have been reviewed in the pair of papers (Chai et al. 2013; Chai and Ngai 2020), with (Chai et al. 2013) analysing 123 papers published from 2008 to 2012, and Chai and Ngai (2020) considering 143 papers from 2013 to 2018. This gives a sense of the number of works published in this area, which makes a full review of these works beyond the scope of this section.

Industry 4.0 is leading to the introduction of new aspects in supplier selection, which are included in recent studies. Sustainability was considered in Giannakis et al. (2020), where the authors developed an Analytic Network Process (ANP) method and used real data collected via extensive surveys from experts in the UK and France. In the last few years, circular manufacturing has been emerging as a novel production paradigm with reduced production waste due to reuse and recycling. A dynamic decision support system (DSS) for sustainable supplier selection in circular manufacturing was proposed in Behrouz et al. (2021) where machine learning is used to maintain the criteria scores after the supplier engagement. Sustainable procurement was studied in Kaur and Singh (2019) which focuses on designing a resilient supply chain with respect to material procurement. They formulated a problem to minimize the overall cost including carbon buying/selling in a trading environment. The suppliers’ flexibility was one of the objectives considered in Bodaghi et al. (2018).

Other work has considered the “green” criterion to evaluate the suppliers, thus representing environmental impact. It includes many factors such as the type of packaging, the reuse of materials and energy, the environmental management system, etc. An AHP-based approach was proposed in Ecer (2020) and includes the evaluation of the suppliers according to green aspects. The authors considered a home appliances manufacturing company as their case study. Similarly, a green supplier evaluation system for a large chemical company was proposed in Bai et al. (2019). Supplier selection has also been considered to reduce the damage caused by natural disasters when relief items are urgently needed in large amounts. The study conducted by Olanrewaju et al. (2020) proposes integrating the supplier selection for the timely distribution of relief supplies. Similarly, another study (Balcik and Ak 2014) tackled the problem from the perspective of organizations in humanitarian relief.

MCDM and mathematical programming methods for supplier selection have been refined and improved in recent years from a methodological perspective. The general trend is to manage incomplete/uncertain data in MCDM by taking into account fuzzy theory, usually by integrating it into standard MCDM approaches. The study by Chang (2019) identifies the best supplier in a supply chain by integrating the intuitionistic fuzzy weighted averaging method and the soft set with imprecise data. A weighted fuzzy multi-objective model that integrates supplier selection, order quantity allocation and scheduling problem was proposed in Bodaghi et al. (2018). Fuzzy Analytic Hierarchy Process (FAHP) strategies were designed in Ortiz Barrios et al. (2020), Kaur and Singh (2021) and Ecer (2020). A general weight-consistent model for supplier selection and order allocation under uncertainty was proposed in Suprasongsin et al. (2019), while a novel interval-valued intuitionistic fuzzy numbers-based reference neighbourhood rough set approach, whose aim is to eliminate the poorest supplier set, was defined in Bai et al. (2019).

The recent works on mathematical programming approaches have different research motivations. A sustainable procurement combinatorial problem was modelled using a Mixed Integer Non-Linear Program (MINLP) in Kaur and Singh (2019). A stochastic multi-objective mathematical model for supplier selection in humanitarian relief was developed in Balcik and Ak (2014). Some studies are devoted to strengthening the MILP formulations by exploiting particular properties. As an example, model improvements to formulate non-linear discounts in terms of MILP were discussed in Andrade-Pineda et al. (2017). Furthermore, a MILP model with some specific valid inequalities and a MILP heuristic was developed for a multi-item inventory lot-sizing problem with supplier selection in Cárdenas-Barrón et al. (2021). Goal programming has also been used to handle multiple objectives in supplier selection by solving a mathematical program, such as in Taleizadeh et al. (2009) where the problem considered is to be a multi-product, multi-constraint, bi-objective newsboy problem with discounts. A few recent mathematical programming approaches manage data uncertainty by formulating the problem in terms of stochastic programming. A p-robust supply chain network design with uncertain demand and cost scenarios, where the supplier selection is integrated with the facility location and capacity problem, was studied in Tian and Yue (2014). The approach proposed in Balcik and Ak (2014) for humanitarian relief is a stochastic programming approach based on different scenarios and minimising the expected cost. Two stochastic models for optimal order allocation, whose uncertainty lies in both the supply and the demand, were proposed in Ray and Jenamani (2016). In He et al. (2009), the authors consider a multiobjective supplier selection problem and convert it into a single objective, non-linear chance-constrained programming problem. A multi-stage stochastic programming approach for supplier selection, which models different types of natural disasters, was presented in Olanrewaju et al. (2020).

Exact approaches based on mathematical programming have a tendency not to be scalable when the problem modelled is NP-hard. This often happens in supplier selection, and the best supplier configuration is computed using means of metaheuristics approaches. Genetic algorithms for supplier selection have been, for example, designed by Taleizadeh et al. (2009) and He et al. (2009), while (Alejo-Reyes et al. 2021) proposed a Particle Swarm Optimisation approach and a Differential Evolution approach.

Many recent supplier selection approaches are based on hybridising two or more techniques. As an example, Shaw et al. (2012) hybridizes Fuzzy-AHP and Fuzzy Multi-Objective MILP. Mehdi (2017) mixes ANP, quality function deployment, and a Markov chain. As said in the introduction, the main difference of our approach compared with the state-of-the-art approaches for supplier selection is that the uncertainty of the DM’s utility function is iteratively reduced by asking a pairwise comparison of queries. Regarding the stochasticity of the supplier selection problems, the current framework only takes into account a deterministic problem. Future extensions may consider the inclusion of stochastic aspects in the MILP model.

### AI for preference elicitation

Preference elicitation is the process of assessing the preferences of a DM, which can be used, for example, to recommend an alternative in a decision-making problem. Preference elicitation procedures can be classified as *content-based*, *collaborative filtering* and *knowledge-based* (Lu et al. 2015; Aggarwal et al. 2016). Content-based methods generate recommendations based on their similarities with the past items liked by the same DM. Collaborative filtering recommends items to a DM by considering the preferences of similar DMs. Knowledge-based recommendations are based on the relationships between the DM and items such as constraints and preference relations. Here we adopted the latter approach with a Multiattribute Utility Theory (MAUT) (Raiffa 1968) setting. MAUT is a branch of MCDM theory whose purpose is to support a DM in the process of selecting alternatives evaluated using a fixed number of conflicting criteria. In this context, the DM is assumed to be endowed with a real-valued utility function that evaluates multiattribute alternatives, where an alternative $s'$ is preferred to another alternative $s''$ if and only if $s'$ has a higher value according to the DM’s utility function. This function can then be used for ranking or recommending alternatives to the DM. In this context, preference elicitation is the process of learning such a function. The goal of classical MAUT approaches (Fishburn 1967; Raiffa 1968; Farquhar 1984) is to precisely specify the DM’s utility function through a series of questions to identify some key values of the utility function. However, experiments with real users (Simon 1955; Tversky and Kahneman 1974; Pu et al. 2003) have shown that this process can be a difficult and error-prone task. Furthermore, it is difficult to apply this approach in a combinatorial domain since it can rapidly become expensive in terms of questions for the DM.

From the 1980s onwards, artificial intelligence has been widely applied in MAUT contexts to develop more robust preference elicitation systems. A major division in recent work on preference elicitation is whether a Bayesian model is assumed over the parameters of the utility function (e.g., the set of weights of the weighted sum value function), or if there is a purely qualitative (logical) representation of the uncertainty. Bayesian approaches include, for example, that shown in the work by Chajewska et al. (2000), Boutilier (2002), Viappiani and Boutilier (2010) and Vendrov et al. (2020). Work involving a qualitative uncertainty representation includes that by Boutilier et al. (2006), Braziunas and Boutilier (2007), Montazery and Wilson (2016), Marinescu et al. (2013) and Toffano and Wilson (2020). In particular, qualitative imprecise preference models based on the weighted sum utility function have been considered in work such as that of Salo and Hämäläinen (2010), Marinescu et al. (2012) and Kaddani et al. (2017). Bayesian methods have the advantage of being more robust with respect to inconsistent input preferences at the expense of an increased computational burden. Qualitative methods are in general faster but inconsistent query responses can compromise the quality of the recommendation. This is because the DM’s inputs are translated into hard constraints, reducing the space of the feasible parameters of the utility function. The wrong answer by a DM could exclude the parameters corresponding to the real DM’s preferences. In our framework, we adopted the latter approach since Bayesian methods would be practically infeasible given the computational burden of our MILP model. In particular, we focused on a qualitative approach based on the minimax regret criterion (Wang and Boutilier 2003; Boutilier et al. 2006; Braziunas and Boutilier 2007, 2008). The max regret of an alternative is the worst-case loss in terms of utility units, and the minimax regret criterion is used to recommend an alternative that minimizes worst-case loss among the feasible set of parameters of the utility function. The practical effectiveness of the minimax regret criterion has been proven in works such as that of Wang and Boutilier (2003), Boutilier et al. (2006) and Braziunas (2012), and in particular during a study carried out with real users (Braziunas and Boutilier 2010).

Different approaches have been explored to interact with the DM (see, e.g., Shin and Ravindran 1991), but we have focused on pairwise comparisons of alternatives to simplify the interaction with the DM. In the literature, there are several methods for query selection based on geometric considerations on the feasible set of parameters of the utility function (Iyengar et al. 2001; Ghosh and Kalagnanam 2003; Toubia et al. 2004; Teso et al. 2016). However, these methods require a normalization of the objectives, which is not a straightforward task in our context (see the discussion at the end of Sect. 5.1). A different approach was proposed in Viappiani and Boutilier (2009) where the authors introduce the concept of setwise max regret that can be used to evaluate the worst-case loss of a set of alternatives with respect to the feasible weights of the utility function. This measure can also be used to evaluate comparison queries defined as a set of alternatives. In fact, the set of alternatives that minimizes the setwise max regret is a myopically optimal query set with respect to the minimax regret criterion (Viappiani and Boutilier 2020). This makes the use of this measure compelling in our framework since we recommend alternatives with respect to the minimax regret criterion. However, the computation of such a query is demanding. Therefore we propose two new methods for query selection based on a novel measure that we call discrepancy. These methods are much faster than evaluating the setwise max regret of all possible query sets, and our experimental results show a similar number of iterations with the DM that were used to achieve convergence.

## Problem requirements

The problem requirements for which our framework is designed come from a real-world study. More specifically, we interacted with the supply chain management of a medium-sized manufacturing factory by asking for information about their internal supplier selection process. As a result of this interaction, we defined a deterministic combinatorial optimization problem with a set of supplier evaluation criteria and constraints. The instances considered in Sect. 6 were artificially generated but they are aligned with the real-world scenario presented.

Given a certain time horizon, the problem consists of computing the quantities to be ordered from each supplier to satisfy the demand for each required component. The upper and lower limits on the number of suppliers per component are considered to be an input. This relates to the fact that the DM may want to have a number of backup suppliers in case of unexpected disruptions. A suppliers’ catalogue is provided as an input as well, including the availability of each component for each supplier and the different prices.

Four different evaluation criteria were considered in the factory supplier selection process. The first criterion considered was cost, including both the direct costs for all of the materials and the activation cost of establishing business relationships with the suppliers. The price breaks (Chaudhry et al. 1993) discount scheme was adopted, meaning that the unit cost is defined depending on how many components of the same type are ordered from the same supplier. This is the standard mechanism adopted by the factory’s suppliers to determine the unit costs for a certain material enquiry. The second and third criteria were the supplier lead time and lateness. They represent the time agreed with a supplier to provide the materials and the lateness with respect to the due date, respectively. The last criterion was supplier reputation. This is a score assigned by internal experts to each supplier upon by considering different aspects such as disruption risk, the relationship between the company and the supplier, and the strategic vision of the firm.

The solutions were evaluated with a utility function defined as the weighted sum of the four evaluation criteria, where the (unknown) weights define the DM’s preferences. Our goal was to define a procedure to find a suitable solution with a low cognitive effort for the DM. Instead of precisely computing the DM’s weights through elaborated interviews as in standard MCDM techniques, we adopted an active learning loop to reduce the uncertainty of the DM’s preferences by asking comparison queries until the max regret of a potential recommendation is below a fixed threshold.

## Terminology and definitions

This section presents some of the key notations used in this paper. Let $\mathcal{P}$ be a combinatorial maximization problem, and let $\mathcal{S}$ be the set of its feasible solutions. Let us define  to be the initial user preferences state space, i.e., the set of all the normalized non-negative weighted vectors $w$. Here, *n* is the number of criteria, so that there is a weight $w_i$ for each criterion *i*. In our supplier selection framework, we consider four criteria, so $n=4$. We consider *n* functions,  $\forall i \in \{1,\dots ,n\}$ over $\mathcal{S}$ and define the vector $g(s) = (g_1 (s), \dots , g_n (s))$ as the utility vector of solution $s\in \mathcal{S}$. The scalar utility of $s\in \mathcal{S}$ with respect to $w\in \mathcal {W}_{0}$, i.e., the objective function of $\mathcal{P}$, is given as $w\cdot g(s) = \sum _{i=1}^n w_i g_i (s)$. For weighted vector $w\in \mathcal {W}_{0}$, let $s_w\in \mathcal{S}$ be an optimal solution of $\mathcal{P}$ with respect to $w$, that is, a solution $s_w$ such that $w\cdot g(s_w)\ge w\cdot g(s)$ for any $s\in \mathcal{S}$.

A weighted vector $w\in \mathcal {W}_{0}$ identifies a specific set of trade-offs among the functions $g_i$ to be optimized in $\mathcal{P}$. Thus, given two solutions $s',s''\in \mathcal{S}$ and a weighted vector $w\in \mathcal {W}_{0}$, $s'$ is at least as good as $s''$ with respect to $w$, if and only if $w\cdot g(s')\ge w\cdot g(s'')$, i.e., $w\cdot (g(s')-g(s''))\ge 0$. This indicates that the scalar utility of the solution $s'$ with respect to $w$ is at least as good as the scalar utility of the solution $s''$ with respect to $w$.

Let $V_\varLambda$ be a convex polyhedron in  defined using a set of non-strict linear inequalities $\varLambda$; we define ${\mathcal{W}_\varLambda }$ as the convex and closed (and thus compact) polytope ${\mathcal{W}_\varLambda }= \mathcal {W}_{0}\cap V_\varLambda$. The linear inequalities in $\varLambda$ can arise from the input preferences of the form $s'$ *is preferred to* $s''$, leading to the constraint $w\cdot (g(s')-g(s'')) \ge 0$.

Let $\textit{Ext}({\mathcal{W}_\varLambda })$ be the set of extreme points of a user preference state space ${\mathcal{W}_\varLambda }$. For each extreme point $w$ we choose an optimal solution $s_w$, and we define $\mathcal{X}_\varLambda$ (abbreviated to $\mathcal{X}$) to be the set $\{s_w: w\in \textit{Ext}({\mathcal{W}_\varLambda })\}$. We say that $\mathcal{X}$ *is a set of optimal solutions with respect to* $\textit{Ext}({\mathcal{W}_\varLambda })$ (given the constraints represented by $\varLambda$).

## The structure of the framework

The main novelty of our supplier selection framework is the fact that the importance of each criterion is defined using a series of interactions with the user, with an interleaving of the elicitation and optimization. In this way, the user drives the solution process in order to reduce the uncertainty with respect to the DM’s trade-offs among the objectives. We define the combinatorial optimization problem $\mathcal{P}$ using the MILP model in Sect. 5.1 below. As in the previous section, let $\mathcal{S}$ be the set of all the feasible solutions of $\mathcal{P}$. The objective function considered in $\mathcal{P}$ is a weighted sum of four functions ${f}_1(s)$, ${f}_2(s)$, ${f}_3(s)$, ${f}_4(s)$, associating a measure of the cost, lateness, lead time and reputation with a feasible solution $s\in \mathcal{S}$ . The analytic form of these functions is described in Sect. 5.1.

The weighted sum used as the objective function of $\mathcal{P}$ is $-w_1 {f}_1(s) - w_2 {f}_2(s) - w_3 {f}_3(s) + w_4 {f}_4(s)$, where $w_i \in [0,1]$ (for each $i \in \{1,2,3,4\}$) is the weight of the *i*th function. The first three signs are negative because the first three functions have to be minimized, whereas ${f}_4(s)$ has to be maximized. The parameters of the MILP model come from different sources. Data like the tariffs and the components’ availability for each supplier comes from the supplier catalogue. On the other hand, the demand for the components are given by an external demand predictor which is not discussed in this paper. Finally, a lateness/lead time predictor is used to predict supplier performances, providing coefficients to be used in ${f}_2(s)$ and ${f}_3(s)$. The predictions are calculated from a database of component orders, containing a series of past orders. The predictor and database of the past orders used in the framework are described in “Appendixs C” and “B”, respectively.

**Fig. 2:**
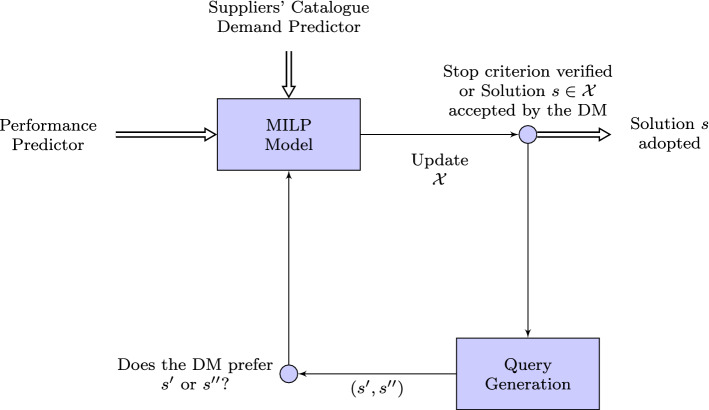
Structure of the proposed framework


The aim of the learning loop described in Fig. 2 is (ideally) to compute an optimal solution $s_{w^*}$ to the combinatorial problem associated with the decision-maker’s unknown preferences, indicated by the vector $w^*=(w^*_1, w^*_2, w^*_3, w^*_4) \in \mathcal {W}_{0}$. As an example, if a decision-maker only cares about minimize the cost, then the associated weighted vector will be (1, 0, 0, 0). In this case, it is easy to define $w^*$ *a priori*, but more typically, the trade-offs among the objectives are harder to define. In general, the precise definition of the preference vector $w$ using standard MCDM methods is liable to be a difficult and error-prone task. As we said in the introduction, the framework therefore uses an alternative approach based on reducing the uncertainty of the DM’s preferences by iteratively asking simple pairwise comparison queries.

Let us consider, as a query $\mathcal{Q}$, a subset of $\mathcal{S}$, associated with a question of the form: which solution do you prefer among the solutions in $\mathcal{Q}$? In our framework, we used queries of the form $\mathcal{Q}=\{s', s''\}$ to learn about $w^*$. This query amounts to do you prefer solution $s'$ or $s''$? The answer implies an inequality of the type $w\cdot g(s')\ge w\cdot g(s'')$ or $w\cdot g(s')\le w\cdot g(s'')$, depending on the DM’s preference between $s'$ and $s''$. In each iteration of the framework, $\varLambda$ is the polyhedron defined as the set of inequalities derived from the user’s answer to the queries presented. These inequalities reduce the user preference space state $\mathcal {W}_{0}$ to ${\mathcal{W}_\varLambda }$, as indicated in Sect. 4. The set $\mathcal{S}$ will tend to be huge, so it will not be feasible to compute it explicitly. The framework makes use of the set $\mathcal{X}$ of optimal solutions associated with the extreme points of ${\mathcal{W}_\varLambda }$, as defined in the last section.

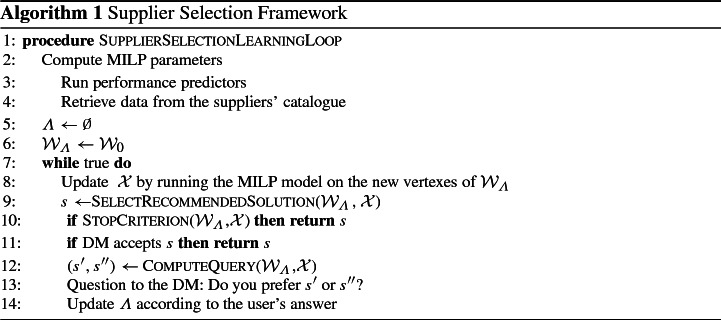


The following lines describe how the framework works in practice, referring to the block diagram in Fig. 2 and the pseudocode depicted in Algorithm 1. The first step is to execute the performance predictors in order to compute the lateness and lead time estimation for each supplier (line 3 of Algorithm 1). The components’ cost and availability per supplier need to be retrieved from the suppliers’ catalogue (line 4). These are input parameters for the MILP model described below in Sect. 5.1.

The next step is to initialize the set of constraints $\varLambda$ to $\emptyset$ and thus ${\mathcal{W}_\varLambda }$ to $\mathcal {W}_{0}$ (lines 5 and 6). The MILP model is then solved for each extreme weighted vector $w \in \textit{Ext}({\mathcal{W}_\varLambda })$ (line 8). When line 8 is executed for the first time, the combinatorial problem is solved four times by optimizing it with respect to each single function ${f}_i(s)$, $i = 1,\dots ,4$; this is because the extreme points of $\mathcal {W}_{0}$ are (1, 0, 0, 0), (0, 1, 0, 0), (0, 0, 1, 0) and (0, 0, 0, 1). Recall $\mathcal{X}$ is the set of solutions generated, following the definition in Sect. 4. A solution $s\in \mathcal{X}$ is selected from those generated through the means of the function SelectRecommendedSolution ${\mathcal{W}_\varLambda },\mathcal{X}$ called at line 9. A stopping criterion is then checked by calling the function StopCriterion ${\mathcal{W}_\varLambda }$,$\mathcal{X}$ (line 10) which determines if ${\mathcal{W}_\varLambda }$ allows one to recommend a solution with a worst-case loss below a certain threshold. If the function returns true, the solution $s$ is provided as an output. Otherwise, we show to the DM the solution $s$ (line 11). If the DM accepts the solution, we stop the algorithm. If the DM is not happy with the solution proposed, a pair of solutions $s',s''\in \mathcal{X}$ is chosen using a user-preference elicitation strategy, implemented by the function ComputeQuery ${\mathcal{W}_\varLambda }$,$\mathcal{X}$ (line 12). The DM then answers the following question (line 13): Do you prefer solution $s'$ or solution $s''$? The answer is used to reduce the uncertainty of the DM’s preferences by updating $\varLambda$ and recomputing ${\mathcal{W}_\varLambda }$ (lines 13–14). In this stage, line 8 is executed again by considering the updated $\varLambda$ and ${\mathcal{W}_\varLambda }$ and the MILP model will run on the extreme points of ${\mathcal{W}_\varLambda }$ that have not been considered in the previous iterations.

As shown in Fig. 2, the main blocks of the framework are the MILP model and the query generation. Sections 5.1 and 5.2 describe these two blocks, with Sect. 5.2 also including the description of the functions SelectRecommendedSolution ${\mathcal{W}_\varLambda },\mathcal{X}$, ComputeQuery ${\mathcal{W}_\varLambda }$,$\mathcal{X}$ and StopCriterion ${\mathcal{W}_\varLambda }$,$\mathcal{X}$.

### The mixed integer linear programming model

Let us consider a set of suppliers ${\mathrm{I}}$ and a set of components ${\mathrm{C}}$. A set of components ${\mathrm{C}}_{i}$ is defined for each supplier ${i}\in {\mathrm{I}}$, consisting of all of the components $j\in {\mathrm{C}}$ that can be provided by supplier ${i}$. The unit cost for a component from a supplier depends on the quantity bought, so multiple unit costs are provided by each supplier. A unit cost is associated with a certain quantity interval, meaning that the unit cost is the same for any quantity in the interval. The set ${\mathrm{T}}_{{i},j}$ is the set of all the disjoint quantity intervals for supplier ${i}\in {\mathrm{I}}$ and component $j\in {\mathrm{C}}_{i}$, whose union covers the set ${\mathbb {N}}$ of positive whole numbers. Let us define the parameter ${m}_{{i},j,{t}} \in {\mathbb {N}}$ as the minimum amount of component $j\in {\mathrm{C}}_{i}$ to be ordered from supplier ${i}\in {\mathrm{I}}$ in the quantity interval ${t}\in {\mathrm{T}}_{{i},j}$. Consequently, ${\mathrm{T}}_{{i},j} = (\cup _{{t}=1}^{|{\mathrm{T}}_{{i},j}|-1} [{m}_{{i},j,{t}}, {m}_{{i},j,{t}+1}-1]) \cup [{m}_{{i},j,|{\mathrm{T}}_{{i},j}|}, +\infty )$, where ${m}_{{i},j,1}=0$. The unit cost associated with a quantity interval ${t}\in {\mathrm{T}}_{{i},j}$ defines a certain tariff and it is indicated with ${c}_{{i},j,{t}}$. The value ${a}_{{i}} \in {\mathbb {R}}_+$ indicates the activation cost of a supplier ${i}\in {\mathrm{I}}$. Note that all the parameters mentioned so far, regarding components cost and availability, come from the suppliers’ catalogue for the factory.

The parameters ${l}_{{i},j,{t}} \in {\mathbb {R}}_+$ and ${\delta }_{{i},j,{t}} \in {\mathbb {R}}_+$ represent respectively the expected lead time and the expected lateness of component $j\in {\mathrm{C}}_{i}$ ordered from ${i}\in {\mathrm{I}}$ in the quantity interval ${t}\in {\mathrm{T}}_{{i},j}$. These parameters are computed by the lateness/lead time predictor. The value ${r}_{j} \in \{1,\ldots ,100\}$ is the reputation of supplier ${i}\in {\mathrm{I}}$. This value is assigned by internal experts, as mentioned in Sect. 3. The values ${\lambda }_{j,min}, {\lambda }_{j,max} \in {\mathbb {N}}$ are the bounds on the number of suppliers for component $j\in {\mathrm{C}}$. Finally, ${D}_j\in {\mathbb {N}}$ is the estimated demand of component $j\in {\mathrm{C}}$.

Our MILP model is based on the following integer decision variables:

- ${x}_{{i},j,{t}} \in {\mathbb {N}}$ is the number of components $j\in {\mathrm{C}}_{i}$ ordered from supplier ${i}\in {\mathrm{I}}$ in the quantity interval ${t}\in {\mathrm{T}}_{{i},j}$;
- ${y}_{{i},j,{t}} \in \{0,1\}$ is equal to 1 if a positive quantity of component $j\in {\mathrm{C}}_{i}$ is ordered from ${i}\in {\mathrm{I}}$ in the quantity interval ${t}\in {\mathrm{T}}_{{i},j}$, and equals 0 otherwise;
- ${z}_{{i}} \in \{0,1\}$ is equal to 1 if at least one component is ordered from the supplier ${i}\in {\mathrm{I}}$, and equals 0 otherwise;
- ${\gamma }_1, {\gamma }_2, {\gamma }_3, {\gamma }_4 \in {\mathbb {R}}_+$ are auxiliary variables used to model the min-max/max-min formulations of the objectives.

Note that the variables ${x}_{{i},j,{t}}$ and ${y}_{{i},j,{t}}$ have three indexes in order to take into account the different costs, lead time and lateness for each triple of supplier ${i}$, component $j$ and quantity interval ${t}$.

A feasible solution $s\in \mathcal{S}$ is determined by a feasible assignment to all these variables. The four functions ${f}_1(s)$, ${f}_2(s)$, ${f}_3(s)$, ${f}_4(s)$ are defined as follows. First, the cost is computed as:

1 $$\begin{aligned} {f}_1(s) = \sum _{{i}\in {\mathrm{I}}, \, j\in {\mathrm{C}}, \, {t}\in {\mathrm{T}}_{{i},j}} {c}_{{i},j,{t}} {x}_{{i},j,{t}} + \sum _{{i}\in {\mathrm{I}}} {a}_{i}{z}_{{i}}. \end{aligned}$$

Both direct costs and the suppliers’ activation costs are taken into account. The goal is to minimize this quantity. The second and third objectives are:

2 $$\begin{aligned} {f}_2(s) = \max _{{i}\in {\mathrm{I}}, \, j\in {\mathrm{C}}_{i}} \sum _{{t}\in {\mathrm{T}}_{{i},j}} {l}_{{i},j,{t}} {y}_{{i},j,{t}}; \end{aligned}$$

3 $$\begin{aligned} {f}_3(s) = \max _{{i}\in {\mathrm{I}}, \, j\in {\mathrm{C}}_{i}} \sum _{{t}\in {\mathrm{T}}_{{i},j}} \delta _{{i},j,{t}} {y}_{{i},j,{t}}. \end{aligned}$$

They represent the maximum expected lead time and the maximum expected lateness related to a certain component and supplier, which are considered to be measures of the quality of service. We want to minimize these quantities. The fourth objective is

4 $$\begin{aligned} {f}_4(s) = \min _{{i}\in {\mathrm{I}}: {z}_{i}=1} {r}_{i}, \end{aligned}$$

which we want to maximize since it indicates the minimum reputation among the suppliers considered in the solution.

The complete MILP model is as follows:

5 $$\begin{aligned}&\max - w_1 {\gamma }_1 - w_2 {\gamma }_2 - w_3 {\gamma }_3 + w_4 {\gamma }_4&\end{aligned}$$

6 $$\begin{aligned} \sum _{{i}\in {\mathrm{I}}} \sum _{{t}\in {\mathrm{T}}_{{i},j}} {x}_{{i},j,{t}}&\ge {D}_j\quad \forall j\in {\mathrm{C}} \end{aligned}$$

7 $$\begin{aligned} {x}_{{i},j,{t}}&\ge {m}_{{i},j,{t}} y_{{i},j,{t}} \quad \forall {i}\in {\mathrm{I}}, \forall j\in {\mathrm{C}}, \forall {t}\in {\mathrm{T}}_{{i},j} \end{aligned}$$

8 $$\begin{aligned} {x}_{{i},j,{t}}&\le M_2 {y}_{{i},j,{t}} \quad \forall {i}\in {\mathrm{I}}, \forall j\in {\mathrm{C}}, \forall {t}\in {\mathrm{T}}_{{i},j} \end{aligned}$$

9 $$\begin{aligned} \sum _{{t}\in {\mathrm{T}}_{{i},j}} {y}_{{i},j,{t}}&\le 1 \quad \forall j\in {\mathrm{C}}, \forall {i}\in {\mathrm{I}} \end{aligned}$$

10 $$\begin{aligned} \sum _{{i}\in {\mathrm{I}}, {t}\in {\mathrm{T}}_{{i},j}} {y}_{{i},j,{t}}&\ge {\lambda }_{j,min} \quad \forall j\in {\mathrm{C}} \end{aligned}$$

11 $$\begin{aligned} \sum _{{i}\in {\mathrm{I}}, {t}\in {\mathrm{T}}_{{i},j}} {y}_{{i},j,{t}}&\le {\lambda }_{j,max} \quad \forall j\in {\mathrm{C}} \end{aligned}$$

12 $$\begin{aligned} {\gamma }_1&= \sum _{j\in {\mathrm{C}}, {i}\in {\mathrm{I}}, {t}\in {\mathrm{T}}_{{i},j}} {c}_{{i},j,{t}} {x}_{{i},j,{t}} + \sum _{{i}\in {\mathrm{I}}} {a}_{i}{z}_{{i}} \end{aligned}$$

13 $$\begin{aligned} {\gamma }_2&\ge \sum _{{t}\in {\mathrm{T}}_{{i},j}} {l}_{{i},j,{t}} {y}_{{i},j,{t}} \quad \forall j\in {\mathrm{C}}, \forall {i}\in {\mathrm{I}} \end{aligned}$$

14 $$\begin{aligned} {\gamma }_3&\ge \sum _{{t}\in {\mathrm{T}}_{{i},j}} {\delta }_{{i},j,{t}} {y}_{{i},j,{t}} \quad \forall j\in {\mathrm{C}}, \forall {i}\in {\mathrm{I}} \end{aligned}$$

15 $$\begin{aligned} {\gamma }_4&\le M_2 (1 - {z}_{i}) + {r}_{i}{z}_i \quad \forall {i}\in {\mathrm{I}} \end{aligned}$$

16 $$\begin{aligned} \sum _{j\in {\mathrm{C}}, {t}\in {\mathrm{T}}_{{i},j}} {y}_{{i},j,{t}}&\le M_3 {z}_{{i}} \quad \forall {i}\in {\mathrm{I}} \end{aligned}$$

17 $$\begin{aligned} \sum _{j\in {\mathrm{C}}, {t}\in {\mathrm{T}}_{{i},j}} {y}_{{i},j,{t}}&\ge {z}_{{i}} \quad \forall {i}\in {\mathrm{I}} \end{aligned}$$

where $M_1,M_2,M_3 \in {\mathbb {R}}_+$ are large enough (“big-*M*”) constants and the other variables/parameters are defined previously. The objective function (5) is the weighted sum of the auxiliary variables ${\gamma }_1,{\gamma }_2, {\gamma }_3, {\gamma }_4$, where the signs are minus for the functions being minimized and plus for the one being maximized. Constraint (6) imposes the condition that the demand per part has to be satisfied. Constraints (7) and (8) are linking constraints between ${x}_{{i},j,{t}}$ and ${y}_{{i},j,{t}}$, which state that ${y}_{{i},j,{t}}$ is active if and only if ${x}_{{i},j,{t}}$ is greater than the minimum quantity ${m}_{{i},j,{t}}$ to unlock the tariff. Constraint (9) forces it so then only one tariff is used when we order a certain quantity from a supplier. Constraints (10) and (11) impose the bounds on the number of suppliers to be selected for each component. Constraint (12) links ${\gamma }_1$ with the analytical expression of ${f}_1(s)$. Constraints (13) and (14) are used for the min-max formulations, so then the auxiliary variables ${\gamma }_2,{\gamma }_3$ are linked to ${f}_2(s),{f}_3(s)$ when the model is solved. Similarly, constraint (15) is used for the max-min formulation regarding ${f}_4(s)$. Expression $M_2 (1 - {z}_{i}) + {r}_{i}{z}_{i}$ is equal to ${r}_{i}$ in the case where the supplier is selected, and equal to $M_2$ otherwise, meaning that the constraint (15) is disabled in the latter case. This expression is then linked to ${\gamma }_4$. Finally, constraints (16) and (17) are the linking constraints among ${y}_{{i},j,{t}}$ and ${z}_{{i}}$, imposing that a certain supplier is active if and only if one component is ordered from it.

In standard interactive preference elicitation models, it is common to normalize the objectives. In this case, this is not a straightforward operation since normalization requires the minimum and the maximum value of each objective, and we are dealing with a combinatorial problem. One could consider maximize and minimize each objective, but $-{\gamma }_2$, $-{\gamma }_3$ and ${\gamma }_4$ are not bounded from below with our problem formulation. Computing an upper bound of the cost maximize ${\gamma }_1$ does not make much sense since in our model, the quantity ordered of each component is bounded from above by an arbitrary big number $M_2$.

The normalization of the objectives can be useful to maintain a similar scale for the weights representing the DM’s preferences with respect to the evaluation criteria. This is very important for query selection strategies based on geometric consideration of the polytope representing the user preferences such as Iyengar et al. (2001), Ghosh and Kalagnanam (2003), Toubia et al. (2004) and Teso et al. (2016). In our framework, we adopted query selection strategies based on the regret of the whole utility function, therefore such rescaling is not essential.

### User-preference elicitation approach

A key point for a good user experience is to reduce the number of interactions with the user by asking informative queries. In this section, we define the different strategies used for the query generation in order to study their impact on the number of iterations required by the framework to converge towards a stopping criterion. Sections 5.2.1 and 5.2.2 introduce some of the preliminary concepts. Section 5.2.3 presents the different query generation strategies, each corresponding with a different implementation of the function ComputeQuery ${\mathcal{W}_\varLambda }$,$\mathcal{X}$ mentioned in Algorithm 1. Section 5.2.4 defines the stopping criterion used in the framework, which is the implementation of StopCriterion ${\mathcal{W}_\varLambda }$,$\mathcal{X}$.

#### Maximum regret

Applying the standard definition, the maximum regret of a feasible solution $s\in \mathcal{S}$ with respect to the user preference state space ${\mathcal{W}_\varLambda }$ is given by:

18 $$\begin{aligned} \textit{MR}_{{\mathcal{W}_\varLambda }}(s,\mathcal{S})= \max _{s'\in \mathcal{S}}\max _{w\in {\mathcal{W}_\varLambda }} w\cdot (g(s')-g(s)). \end{aligned}$$

Intuitively, $\textit{MR}_{{\mathcal{W}_\varLambda }}(s,\mathcal{S})$ represents the worst-case loss due to recommending the solution $s$ with respect to the user preference state space ${\mathcal{W}_\varLambda }$ and all of the possible recommendations $s'\in \mathcal{S}$. Note that $\textit{MR}_{{\mathcal{W}_\varLambda }}(s,\mathcal{S})\ge 0$ since $s\in \mathcal{S}$, and $s'=s$ gives $w\cdot (g(s')-g(s))=0$.

As mentioned earlier, computing the set $\mathcal{S}$ of feasible solutions is not practically feasible. However, the following proposition (based on a well-known property of maximum regret, see e.g., Timonin 2013) allows us to compute the maximum regret of a solution $s\in \mathcal{S}$ with respect to any $w\in {\mathcal{W}_\varLambda }$ and $s'\in \mathcal{S}$ using just the set $\textit{Ext}({\mathcal{W}_\varLambda })$ of extreme points of ${\mathcal{W}_\varLambda }$ and the corresponding set $\mathcal{X}$ of optimal solutions.

##### Proposition 1

Let $\mathcal{S}$ be the set of all the feasible solutions with respect to ${\mathcal{W}_\varLambda }$, let $s$ be an element of $\mathcal{S}$ and let $\mathcal{X}$ be a set of optimal solutions with respect to $\textit{Ext}({\mathcal{W}_\varLambda })$. Then $\textit{MR}_{{\mathcal{W}_\varLambda }}(s,\mathcal{S})=\textit{MR}_{\textit{Ext}({\mathcal{W}_\varLambda })}(s,\mathcal{X})$

##### Proof

${\mathcal{W}_\varLambda }$ is a continuous space but since the scalar utility of a solution $s\in \mathcal{S}$ is a linear function of $w$, the regret of $s$ is maximized on an extreme point of ${\mathcal{W}_\varLambda }$, i.e., $\textit{MR}_{{\mathcal{W}_\varLambda }}(s,\mathcal{S}) = \textit{MR}_{\textit{Ext}({\mathcal{W}_\varLambda })}(s,\mathcal{S})$. Since $\mathcal{X}$ is a set of optimal solutions with respect to $\textit{Ext}({\mathcal{W}_\varLambda })$, then $\textit{MR}_{\textit{Ext}({\mathcal{W}_\varLambda })}(s,\mathcal{S})$ which equals $\max _{s'\in \mathcal{S}}\max _{w\in \textit{Ext}({\mathcal{W}_\varLambda })}w\cdot (g(s')-g(s)) = \max _{s'\in \mathcal{X}}\max _{w\in \textit{Ext}({\mathcal{W}_\varLambda })}w\cdot (g(s')-g(s)) = \textit{MR}_{\textit{Ext}({\mathcal{W}_\varLambda })}(s,\mathcal{X})$. $\square$

The argument returned by SelectRecommendedSolution ${\mathcal{W}_\varLambda },\mathcal{X}$ (a method defined in Algorithm 1) will be a solution $s\in \mathcal{X}$ that minimizes $\textit{MR}_{\textit{Ext}({\mathcal{W}_\varLambda })}(s,\mathcal{X})$.

The concept of maximum regret can be extended in a setwise sense in order to evaluate the worst-case loss of a set of solutions (Viappiani and Boutilier 2009, 2011). Let $\textit{Val}_\mathcal{S}(w)$ be $\max _{s\in \mathcal{S}} w\cdot g(s)$ (the maximum scalar utility we can get from solutions $s\in \mathcal{S}$ assuming that the weighted vector is $w\in {\mathcal{W}_\varLambda }$). The setwize maximum regret (SMR) for a subset $\mathcal{Q}\subseteq \mathcal{S}$ with respect to the user preference state space ${\mathcal{W}_\varLambda }$ is then defined as:

19 $$\begin{aligned} \begin{aligned} \textit{SMR}_{{\mathcal{W}_\varLambda }}(\mathcal{Q}, \mathcal{S})&=\max _{s'\in \mathcal{S}}\max _{w\in {\mathcal{W}_\varLambda }} (w\cdot g(s')- \max _{s\in \mathcal{Q}}w\cdot g(s))\\&= \max _{w\in {\mathcal{W}_\varLambda }}(\textit{Val}_{\mathcal{S}}(w)-\textit{Val}_\mathcal{Q}(w)). \end{aligned} \end{aligned}$$

Intuitively, the SMR of a set $\mathcal{Q}\subseteq \mathcal{S}$ represents the worst-case loss of $\mathcal{Q}$ with respect to the user preference state space ${\mathcal{W}_\varLambda }$ and the set of possible solutions $\mathcal{S}$.

Note that in this case we are evaluating a set rather than a single element. This means that the setwise maximum regret cannot be computed by considering only the extreme points of ${\mathcal{W}_\varLambda }$. In order to consider the whole user preference state space ${\mathcal{W}_\varLambda }$, the value $\textit{SMR}_{{\mathcal{W}_\varLambda }}(\mathcal{Q}, \mathcal{S})$ can be computed as $\max _{s'\in \mathcal{S}}\textit{SMR}_{{\mathcal{W}_\varLambda }}(\mathcal{Q}, \{s'\})$. Each sub-problem $\textit{SMR}_{{\mathcal{W}_\varLambda }}(\mathcal{Q}, \{s'\})$ can be computed, using a linear programming solver, as the maximum value of real variable $\alpha$ subject to a constraint $w\cdot (g(s')-g(s))\ge \alpha$ for each $s\in \mathcal{Q}$, where $w$ is constrained to lie in ${\mathcal{W}_\varLambda }$.

#### Discrepancy measure

Given $v\in {\mathcal{W}_\varLambda }$, recall from Sect. 4 that $s_{v}\in \mathcal{S}$ is a corresponding optimal solution computed from the discrete optimization problem, we define the *discrepancy* of $s\in \mathcal{S}$ with respect to $v$ as $D_{v}(s) = v\cdot (g(s_{v})-g(s))$. This is a measure of how good the solution $s$ is, assuming that the user weighted vector is $v$. Note that $D_{v}(s)\ge 0$ for any $s'\in \mathcal{S}$ since $s_{v}$ is an optimal solution with respect to$v$, i.e., $v\cdot g(s_{v})\ge v\cdot g(s)$ for any $s\in \mathcal{S}$. We will use this measure to select a query composed of a pair $s_{u},s_v\in \mathcal{X}$ of solutions with high values of $D_{v}(s_{u})$ and $D_{u}(s_v)$. The idea is to ask the user to express a preference between two optimal solutions that are maximally different with respect to the corresponding weighted vectors, in order to reduce as much as possible the uncertainty with respect to the DM’s preferences.

Because the computation of set $\mathcal{S}$ is impractical, we limited our approach to the computation of the set $\mathcal{X}$ of optimal solutions computed by a linear programming solver with respect to the extreme points $\textit{Ext}({\mathcal{W}_\varLambda })$ of the user preference state space ${\mathcal{W}}$. According to Proposition 1, $\textit{MR}_{{\mathcal{W}_\varLambda }}(s,\mathcal{S})=\textit{MR}_{\textit{Ext}({\mathcal{W}_\varLambda })}(s,\mathcal{X}) = \max _{v\in \textit{Ext}({\mathcal{W}_\varLambda })} \max _{s'\in \mathcal{X}}(v\cdot (g(s')-g(s)))$, which can be written as $\max _{v\in \textit{Ext}({\mathcal{W}_\varLambda })}(v\cdot (g(s_v)-g(s)))$. This shows that the maximum regret of a solution can be expressed using the discrepancy function:

20 $$\begin{aligned} \textit{MR}_{{\mathcal{W}_\varLambda }}(s,\mathcal{S})= \textit{MR}_{{\mathcal{W}_\varLambda }}(s,\mathcal{X})= \max _{v\in \textit{Ext}({\mathcal{W}_\varLambda })}D_{v}(s). \end{aligned}$$

#### Query generation

Let $\mathcal{Y}$ be a non-empty subset of $\mathcal{S}$. We say that a solution $s'\in \mathcal{Y}$ is *undominated* in $\mathcal{Y}$ with respect to${\mathcal{W}_\varLambda }$ if there does not exist $s''\in \mathcal{Y}$ such that (i) $w\cdot g(s'')\ge w\cdot g(s')$ for all $w\in {\mathcal{W}_\varLambda }$, and (ii) $w\cdot g(s'')>w\cdot g(s')$ for at least one $w\in {\mathcal{W}_\varLambda }$. We say that $\mathcal{Y}$ is *equivalence-free* with respect to${\mathcal{W}_\varLambda }$ if $\mathcal{Y}$ has no equivalent solutions in ${\mathcal{W}_\varLambda }$, i.e., there are no differing elements $s',s''\in \mathcal{Y}$ such that $w\cdot g(s'')=w\cdot g(s')$ for all $w\in {\mathcal{W}_\varLambda }$. We say that a query $\mathcal{Q}=\{s', s''\}$ is *informative* if the corresponding cut generated by the user answer will reduce the user preference state space, regardless of which answer is received, i.e., if there exists $u,v\in {\mathcal{W}_\varLambda }$ such that $u\cdot g(s')>u\cdot g(s'')$ and $v\cdot g(s'')>v\cdot g(s')$.

##### Proposition 2

If a set of solutions $\mathcal{Y}$ is equivalence-free and it contains only undominated elements, then any query $\mathcal{Q}=\{s', s''\}$ with $s',s''\in \mathcal{Y}$ and $s'\ne s''$ is informative.

##### Proof

Consider any query $\mathcal{Q}=\{s', s''\}$ with $s',s''\in \mathcal{Y}$ and $s'\ne s''$. Since $\mathcal{Y}$ only contains undominated elements and is equivalence-free, we have that $s''$ does not dominate $s'$ and is not equivalent to it. So there exists $u\in {\mathcal{W}_\varLambda }$ with $u\cdot g(s')>u\cdot g(s'')$. Similarly, there exists $v\in {\mathcal{W}_\varLambda }$ with $v\cdot g(s'')>v\cdot g(s')$, showing that $\mathcal{Q}=\{s', s''\}$ is an informative query. $\square$

For example, with $\textit{Ext}({\mathcal{W}_\varLambda })=\{u=(1,0,0), \ v=(0.5,0.5,0), \ t=(0,0,1)\}$ and $\mathcal{X}=\{s_u=(2,0,2), \ s_v=(2,2,0), \ s_t=(0,2,2)\}$), if we select the query $\mathcal{Q}=\{s_{u}, s_{t}\}$ and the user answer is $s_{u}$, then the cut $w\cdot g(s_{u})\ge w\cdot g(s_{t})$ will not reduce the space ${\mathcal{W}_\varLambda }$. The solution $s_{t}$ is dominated by $s_{u}$ since $u\cdot g(s_{u})>u\cdot g(s_{t})$, $v\cdot g(s_{u})=v\cdot g(s_{t})$ and $t\cdot g(s_{u})=t\cdot g(s_{t})$. Therefore if we had first removed the dominated elements of $\mathcal{X}$ then the query $\mathcal{Q}=\{s_{u}, s_{t}\}$ could not have been selected.

Let $\textit{UD}_{\mathcal{W}_\varLambda }(\mathcal{X})$ be the set of undominated solutions of $\mathcal{X}$ with respect to${\mathcal{W}_\varLambda }$ (which is always non-empty). Note that $\textit{UD}_{\mathcal{W}_\varLambda }(\mathcal{X})=\textit{UD}_{\textit{Ext}({\mathcal{W}_\varLambda })}(\mathcal{X})$ since the scalar utility of a solution is a linear function with respect to$w\in {\mathcal{W}_\varLambda }$. We can compute $\textit{UD}_{\mathcal{W}_\varLambda }(\mathcal{X})$ and at the same time make $\mathcal{X}$ equivalence-free as follows. If it is the case that $w\cdot (g(s')-g(s''))= 0$ for all $w\in \textit{Ext}({\mathcal{W}_\varLambda })$, then we remove either $s'$ or $s''$. We then remove all $s''\in \mathcal{X}$ such that there exists $s'\in \mathcal{X}$ with $w\cdot g(s'')\le w\cdot g(s')$ for all $w\in \textit{Ext}({\mathcal{W}_\varLambda })$.

Once we make $\mathcal{X}$ equivalence-free and devoid of dominated elements, we can proceed with the query selection process. We considered the following three methods to select a query $\mathcal{Q}=\{s_{u}, s_{v}\}$ from $\mathcal{X}$ (with their relative performance being compared in Sect. 6):

1. Setwise min max regret (SMMR): select a query $\mathcal{Q}\subseteq \mathcal{X}$ with $|\mathcal{Q}|=2$ that minimizes $\textit{SMR}_{{\mathcal{W}_\varLambda }}(\mathcal{Q},\mathcal{X})$.
2. Max min discrepancy (MMD): select a query $\mathcal{Q}\subseteq \mathcal{X}$ with $|\mathcal{Q}|=2$ that maximizes $\textit{MMD}(\mathcal{Q})=\min (D_{v}(s_{u}), D_{u}(s_{v}))$.
3. Max discrepancy sum (MDS): select a query $\mathcal{Q}\subseteq \mathcal{X}$ with $|\mathcal{Q}|=2$ that maximizes $\textit{MDS}(\mathcal{Q})= D_{v}(s_{u}) + D_{u}(s_{v}) = (u-v)\cdot (g(s_{v})-g(s_{u}))$.

Each of these methods can be used to implement ComputeQuery ${\mathcal{W}_\varLambda }$,$\mathcal{X}$ used in Algorithm 1.

SMMR combines the quality of the solutions with being maximally informative (Viappiani and Boutilier 2009). This ensures a good diversity of solutions shown to the user. However, computing a query that minimizes the setwise maximum regret is quite expensive since we need to solve the $O(n^3)$ linear programming problems, where $|\mathcal{X}|=n$. This is because we have to evaluate the SMR of each possible query $\mathcal{Q}$, and for each query $\mathcal{Q}$ we need to solve *O*(*n*) linear programming problems (see Sect. 5.2.1). MDS and MMD are two simpler methods that we developed that consider only the two weighted vectors associated with the solutions composing the query rather than the whole user preference state space ${\mathcal{W}_\varLambda }$. The aim is still to be maximally informative but with a lower complexity for the evaluation of each query. In this case, the most expensive operation in the evaluation of a query is the dot product. We can also store and reuse the value of a query for subsequent iterations in cases where the corresponding extreme points are not removed by the preference elicitation process.

A recent paper (Benabbou and Lust 2019) proposed a similar interactive preference elicitation procedure, i.e., the queries for the user are computed using the solutions associated with the extreme points of the polytope representing the preferences learned so far. From the experimental results, it looks like the best method for query selection was *Max-Dist*, i.e., computing the query as the pair of solutions that maximize the corresponding Euclidean distance. During the development of our framework, we considered this method but discarded it since our initial experimental results indicated that it did not perform well compared to the other methods we have presented in this paper. We believe that the poor efficacy of this method applied in our context is due to its high sensitivity to the scales of the objectives of the utility function to be optimized. In fact, this method is designed for an objective function with normalized evaluation criteria, but such a normalization is not feasible for our problem formulation (see the end of Sect. 5.1). Note that the idea behind our MDS method is somewhat similar, since we selected a pair of solutions that maximize $(u-v)\cdot (g(s_{v})-g(s_{u}))$, i.e., the dot product between (i) the difference between the corresponding weighted vectors, and (ii) the difference of the utilities of the corresponding extreme points. It may well be that MDS performs better in our context because it is much less sensitive to any changes in the particular choice of utility scales.

#### Stopping criterion

Let ${\mathrm{NO}}_{\mathcal{W}_\varLambda }(\mathcal{S})$ be the set of the *necessarily optimal* solutions of $\mathcal{S}$ with respect to${\mathcal{W}_\varLambda }$, i.e., the set of solutions $s'\in \mathcal{S}$ such that $w\cdot (g(s')-g(s''))\ge 0$ for any $s''\in \mathcal{S}$ and for any $w\in {\mathcal{W}_\varLambda }$. These are the solutions that are optimal with respect to every consistent weighted vector. Note that usually there are no necessarily optimal solutions, unless ${\mathcal{W}_\varLambda }$ is a small set. Also, if there is more than one necessarily optimal element, they are all equivalent. If there exists a solution $s'\in \mathcal{S}$ such that $D_{v}(s')=0$ for all $v\in \textit{Ext}({\mathcal{W}_\varLambda })$, since ${\mathcal{W}_\varLambda }$ is a convex and compact set, there is no solution better than $s'$ with respect tothe user preference state space ${\mathcal{W}_\varLambda }$, i.e., $s'\in {\mathrm{NO}}_{\mathcal{W}_\varLambda }(\mathcal{S})$. As is well known (see e.g., Timonin 2013 or Bourdache and Perny 2019), $s'\in {\mathrm{NO}}_{\mathcal{W}_\varLambda }(\mathcal{S})$ if and only if $\textit{MR}_{{\mathcal{W}_\varLambda }}(s',\mathcal{S})=0$. These equivalences are expressed more formally by the following proposition.

##### Proposition 3

Let $s\in \mathcal{S}$ be a feasible solution, then the following statements are equivalent:

- $$D_{v}(s)=0$$ for all $$v\in \textit{Ext}({\mathcal{W}_\varLambda })$$;
- $$s\in {\mathrm{NO}}_{{\mathcal{W}_\varLambda }}(\mathcal{S})$$;
- $$\textit{MR}_{{\mathcal{W}_\varLambda }}(s,\mathcal{S})=0$$.

##### Proof

(a)$\Rightarrow$(b): If $D_{v}(s)=0$ for each $v\in \textit{Ext}({\mathcal{W}_\varLambda })$, then $v\cdot (g(s_{v})-g(s)) = 0$ for each $v\in \textit{Ext}({\mathcal{W}_\varLambda })$. Therefore, since $s_{v}$ is an optimal solution with respect to$v$, $s$ is optimal for all $v\in \textit{Ext}({\mathcal{W}_\varLambda })$, then $w\cdot (g(s)-g(s')) \ge 0$ for each $w\in \textit{Ext}({\mathcal{W}_\varLambda })$ and for any $s'\in \mathcal{S}$. Since ${\mathcal{W}_\varLambda }$ is convex and compact, any $w'\in {\mathcal{W}_\varLambda }$ can be expressed as a convex combination of extreme points in $\textit{Ext}({\mathcal{W}_\varLambda })=\{w_1,\ldots ,w_n\}$, i.e., $w'=\sum ^n_{i=1} \lambda _iw_i$ for some $\lambda _i\in [0,1]$ such that $\sum ^n_{i=1}\lambda _i=1$, then $w'\cdot (g(s)-g(s'))=\sum ^n_{i=1}\lambda _iw_i(g(s)-g(s'))\ge 0$, and then $s$ is optimal for any $w'\in {\mathcal{W}_\varLambda }$, i.e., $s\in {\mathrm{NO}}_{\mathcal{W}_\varLambda }(\mathcal{S})$.

(b)$\Rightarrow$(c) If $s\in {\mathrm{NO}}_{\mathcal{W}_\varLambda }(\mathcal{S})$, then $w\cdot (g(s')-g(s))\le 0$ for any $s'\in \mathcal{S}$ and for any $w\in {\mathcal{W}_\varLambda }$. Therefore $\textit{MR}_{{\mathcal{W}_\varLambda }}(s,\mathcal{X})= \max _{s'\in \mathcal{X}}\max _{w\in {\mathcal{W}_\varLambda }}(w\cdot (g(s')-g(s)))\le 0$, but since $\textit{MR}_{{\mathcal{W}_\varLambda }}(s,\mathcal{X})\ge 0$, we have $\textit{MR}_{{\mathcal{W}_\varLambda }}(s,\mathcal{X})=0$.

(c)$\Rightarrow$(a) If $\textit{MR}_{{\mathcal{W}_\varLambda }}(s,\mathcal{X})=0$, since $\textit{MR}_{{\mathcal{W}_\varLambda }}(s,\mathcal{X})=\max _{v\in \textit{Ext}({\mathcal{W}_\varLambda })}D_{v}(s)$ (see Sect. 5.2.2) and $D_{v}(s)\ge 0$ for any $v\in {\mathcal{W}_\varLambda }$, then $D_{v}(s)=0$ for all $v\in \textit{Ext}({\mathcal{W}_\varLambda })$. $\square$

Because of Proposition 3, if we find a solution $s\in \mathcal{X}$ such that $D_{v}(s)=0$ for each $v\in \textit{Ext}({\mathcal{W}_\varLambda })$, we can stop the algorithm and recommend $s$ to the user since it will be an optimal solution with respect to any $w\in {\mathcal{W}_\varLambda }$.

Our iterative procedure is possible to repeat until we find a necessarily optimal solution in $\mathcal{X}$. However, if there are a large set of solutions that are optimal with respect to similar weighted vectors, we might need too engage in too many interactions with the user in order to find a necessarily optimal solution, obtaining only small improvements in each iteration. Because of this, we used, as a stopping criterion, the condition that the minimax regret is small. The minimax regret is zero if and only if there is a necessarily optimal solution. We therefore implemented the function StopCriterion ${\mathcal{W}_\varLambda }$,$\mathcal{X}$ defined in Algorithm 1 as follows. At each iteration we checked the maximum regret of each solution $s\in \mathcal{X}$ and if there is at least one solution with a maximum regret lower than a specific threshold $\epsilon$, we stop the algorithm and recommend the solution with a minimum max regret. Furthermore, in each iteration, we show the solution $s\in \mathcal{X}$ minimize the max regret so then the DM can stop the execution if the proposed solution is good enough.

## Computational experiments

The aim of this section was to assess the computational effectiveness of the framework by considering the three different preference elicitation strategies described in Sect. 5.2.3. Two different performance measures were considered: the number of queries generated and the overall computational time required to reach the stopping criterion. The number of queries generated is equivalent to the number of interactions with the user, which is an important measure of the framework usability. In contrast with the computational time, this performance measure focuses on measuring the quality of the user preferences strategy adopted, and it does not depend on the approach used to solve the combinatorial problem.

The computational experiments were performed on randomly generated instances that represent realistic scenarios as described in Sect. 6.1. Section 6.2 presents the computational results and discusses how the framework performs under different conditions.

### Instances structure

Each instance considered was generated by considering, as an input, the number of suppliers $|{\mathrm{I}}|$, the number of components $|{\mathrm{C}}|$ and the density parameter ${{\rho }}\in {\mathbb {R}}$, where the latter enforces that the total number of pairs (*i*, *j*), (where supplier $i \in {\mathrm{I}}$ can provide component $j \in {\mathrm{C}}$) is equal to ${{\rho }}\cdot |{\mathrm{I}}| \cdot |{\mathrm{C}}|$ rounded to the nearest integer. The component availability of each supplier was randomly assigned such that the overall density ${{\rho }}$ was enforced using the procedure described in “Appendix A”.

The instances were structured in order to reflect a scenario in which the firm needs a large number of low price components and a small number of expensive ones. Bearing this in mind, the set of components ${\mathrm{C}}$ was partitioned into three categories: *Cheap*, *Average* and *Expensive*, which included 75%, 20% and 5% of the overall number of components. The demand ${D}_j$ of each component $j\in {\mathrm{C}}$ depended on its category. It was sampled from a Gaussian distribution with a mean $\mu ^d_j$ and standard deviation $\sigma ^d_j$ (discarding values that are less than or equal to zero), using the values reported in Table 1.

**Table 1:** Mean and standard deviations of the Gaussian distribution used to sample the demand of a component with respect to each category

	Cheap	Average	Expensive
$\mu ^d_j$	3000	600	90
$\sigma ^d_j$	750	150	22.5

The unit cost of each component depends on its category, the supplier and the quantity ordered. An average cost $\mu ^c_j$ per component $j\in {\mathrm{C}}$ was computed by considering a uniform distribution over the interval associated with the component category, as defined in Table 2. The unit cost of a component provided by a supplier ${i}\in {\mathrm{I}}$ was then sampled with a uniform distribution on the interval $[0.9 \mu ^c_j, 1.1 \mu ^c_j]$. Finally, a random discount was considered to compute the costs, by sampling uniformly on the intervals indicated in Table 3, which depended on the quantity ordered. The lower limits on the quantities indicated in the table represent the coefficients ${m}_{{i},j,{t}}$ of Eq. 7. By following the steps described above, the unit cost parameters ${c}_{{i},j,{t}}$ were computed.

**Table 2:** Intervals of the uniform distributions used to sample the mean cost of the components with respect to each category

Cheap	Average	Expensive
[0.05, 3]	[4, 30]	[50, 200]

The activation costs ${a}_{i}$ (for ${i}\in {\mathrm{I}}$) were defined such that the impact on the overall cost function is of the same order of magnitude as the direct costs. The following steps were followed in order to achieve this goal. Let $\mu ^c_{j,TOT}=\sum _{j\in {\mathrm{C}}}{D}_j\cdot \mu ^c_j$ be the average total cost to satisfy the whole demand of all components. Assuming that we rely on only $\frac{|{\mathrm{I}}|}{2}$ suppliers, the average amount of direct costs per supplier is equal to $\frac{2 \mu ^c_{j,TOT}}{|{\mathrm{I}}|}$. We then sampled each activation cost ${a}_{i}$ from a uniform distribution on the interval $[0.8 \frac{2 \mu ^c_{j,TOT}}{|{\mathrm{I}}|}, 1.2 \frac{2 \mu ^c_{j,TOT}}{|{\mathrm{I}}|})$.

**Table 3:** Discount intervals per component category and quantity ordered

Quantity	Discount
*Cheap*	
1–750	0%
751–1000	[3,7]%
1001–1250	[8,12]%
> 1251	[13,17]%
*Average*	
1–150	0%
151–200	[3,7]%
201–250	[8,12]%
> 251	[13,17]%
*Expensive*	
1–20	0%
21–30	[3,7]%
31–40	[8,12]%
> 41	[13,17]%

The parameters ${\lambda }_{j,min}$ (Eq. 10) and ${\lambda }_{j,max}$ (Eq. 11) representing the bounds on the number of components per supplier $j$ were sampled using a discrete uniform distribution on the set of integers $\{1,2\}$ and on $\{{\lambda }_{j,min},\ldots , 5\}$, respectively. The parameters representing the expected lead time ${l}_{{i},j,{t}}$ and expected delay ${\delta }_{{i},j,{t}}$ in Eqs. 13 and 14 were computed using a supplier performance predictor (see “Appendix C”) based on a database of past orders (see “Appendix B”). Finally, the reliability ${r}_{i}$ (Eq. 15) of each supplier ${i}$ was defined by sampling the discrete uniform distribution from the set $\{1, \ldots , 100\}$.

### Experimental results

The framework was implemented in Python 3.7 including the MILP model generation and the different preference elicitation strategies. CPLEX 12.8 (ILOG 2017) was used as a MILP and LP solver, while the Python library pycddlib (Troffaes 2018) was used to compute the extreme points of the user-preference polytope. All of the experiments described below were performed on an Intel(R) Xeon(R) E5620 2.40 GHz processor with 32 GB of RAM.

The instances considered were randomly generated as described in Sect. 6.1. We generated 20 instances for each triple $(|{\mathrm{I}}|, |{\mathrm{C}}|, {{\rho }})$, such that $|{\mathrm{I}}| \in \{10,20,30\}$, $|{\mathrm{C}}| \in \{30,40,50,60\}$ and ${{\rho }}\in \{0.2,0.3,0.4,0.5\}$. As a result, the overall set of instances has $20 \cdot 3 \cdot 4 \cdot 4 = 960$ elements.

Table 4 shows the performance of the different strategies SMMR, MMD and MDS with respect to time and the number of queries. The first three columns of both tables contain the values of the parameters $|{\mathrm{I}}|$, $|{\mathrm{C}}|$ and ${{\rho }}$, while the fourth column gives the percentage $\alpha$ of instances where the convergence to the stopping criterion was achieved within the time limit of 2 hours. The remaining columns show the average $\mu _{time}$ computational time and the average $\mu _{query}$ of the number of queries for each of the proposed strategies. The results reported for the last six columns take into account only the instances where convergence was achieved within the time limit.

We needed a common measure to compare the rows of Table 4 and summarize the performance of the three methods; a simple mean for each column would strongly bias the results towards the larger instances. Instead, for each result (i.e., average time or average number of queries), we computed a score that we called the *ratio with the best method* (RWB), dividing the result by the corresponding best result among the three methods in that row. For example, the RWB value for the SMMR query time for the first row is equal to 1.12/0.81. We then considered the mean of the values over all 48 rows. These values were recorded in the last row of the table.

The 20 instances generated for each triple $(|{\mathrm{I}}|, |{\mathrm{C}}|, {{\rho }})$ have a different unknown user preference vector that was generated randomly by the means of the procedure described below. The first aspect to consider when defining this procedure is the different scales of the four objective functions. For example, a user preference vector $w_u=(0.25, 0.25, 0.25, 0.25)$ does not necessarily describe a case in which the same importance is given to each of the four objectives, since the choice of scales of the objectives can be somewhat arbitrary. Because of the difference in scales, a vector of (0.25, 0.25, 0.25, 0.25) might implicitly give a much higher importance to e.g., the first objective. For this reason, we chose not to sample $w_u$ with a uniform distribution (which could lead to the first objective being the most important one for almost all instances) and instead to use a distribution that gives a higher probability to the more extreme vectors. More precisely, we used the following method:

1. We solved the MILP problem using the extreme points $w1=(1,0,0,0)$, $w2=(0,1,0,0)$, $w3=(0,0,1,0)$ and $w4=(0,0,0,1)$ of the initial weighted vector state space $\mathcal {W}_{0}$, and let $s'_{wi}$ be the solution computed with the weighted vector $wi$ where $i\in \{1,\ldots ,4\}$.
2. We computed the value $k_i=\frac{rnd[0,1)}{s'_{wi}(i) - \min _{j\in \{1, \ldots , 4\}}(s'_{wj}(i))}$ for each $i\in \{1, \ldots , 4\}$.
3. We set the weighted vector to $w_p=\frac{1}{\sum ^4_{j=1} k_j}(k_1, k_2, k_3, k_4)$

The idea is to try to define an approximation of the range of each objective in order to re-scale a random vector with respect to the ranges of the objective functions.

**Fig. 3:**
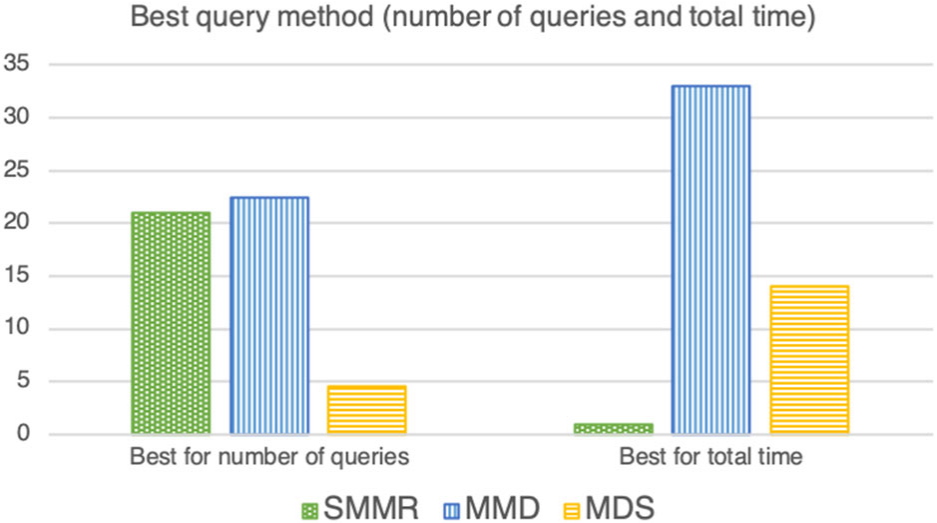
The number of experiments in which the three methods of query selection achieved the best performances with respect to the number of queries and the CPLEX time

The bar chart in Fig. 3 counts the number of times in which each of the three methods used for query selection achieved the best average performance given a triple $(|{\mathrm{I}}|, |{\mathrm{C}}|, {{\rho }})$ of Table 4, with respect to the number of queries and the total computational time. More specifically, the frequency in this bar chart is based on the score given to each strategy. This score is based on summing up 1 unit in the case the strategy is the only method achieving the best performances, a half a unit in the case of a tie between two strategies, and a third of a unit in the case of a three-way tie.

As we can see from Fig. 3 and the last row of Table 4, it looks like that MMD is on average better than the other two methods in terms of the total time and (perhaps surprisingly) the number of queries.

**Fig. 4:**
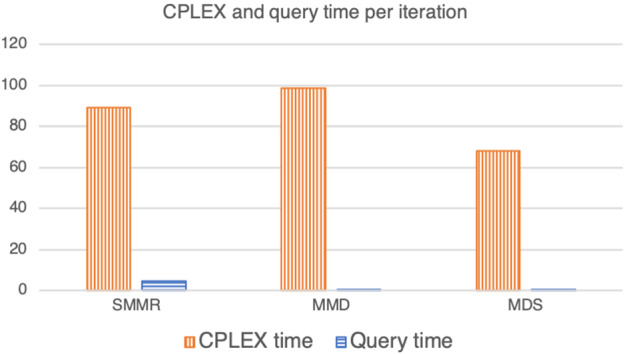
Average CPLEX time per iteration and the average query computation time for the three methods of query selection. The graph shows an average of 20 instances where $|{\mathrm{I}}|=10$, $|{\mathrm{C}}|=30$ and ${{\rho }}=0.4$

**Fig. 5:**
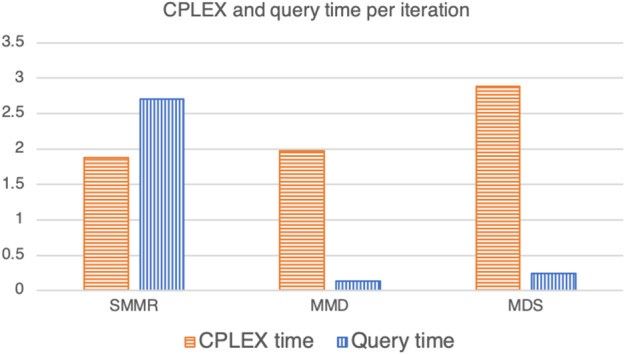
Average CPLEX time per iteration and query computation time for the three methods for query selection. The graph shows an average of 20 instances where $|{\mathrm{I}}|=30$, $|{\mathrm{C}}|=50$ and ${{\rho }}=0.4$

Figures 4 and 5 show the average CPLEX execution time per iteration and the average query computation time per iteration for the three methods of query selection for the two different experiment configurations, i.e., 10 suppliers, 30 components and 0.4 density, and 30 suppliers, 50 components and 0.4 density. The average CPLEX execution time per iteration is computed as the sum of the total CPLEX time for each instance divided by the sum of the number of iterations for each instance. The average query computation time per iteration is computed as the sum of the total query time for each repetition divided by the sum of the total number of queries for each instance. As we can see in Figs. 4 and 5, the query time is much higher for the SMMR method. This is not surprising since SMMR has a higher computational burden than MMD and MDS. It is interesting to see that the choice of the query selection method has a substantial impact on the total time for small instances (see Fig. 4). On the contrary, the time taken by the query selection methods is negligible when the size of the instances is large enough (see Fig. 5).

Generally speaking, the results show that the strategies SMMR and MMD look better than MDS in terms of the number of queries generated. A possible explanation is that the discrepancy sum computed in MDS, which drives the query generation process, can be high even if one of the two solutions in the selected pair $(s_{u},s_v)$ has a discrepancy value that is close to zero. In such a scenario, it may happen that the region of the polytope ${\mathcal{W}_\varLambda }$ in which $w\cdot (g(s_v)-g(s_u))\ge 0$ holds is very small. Therefore, if the user prefers $s_u$ to $s_v$, the cut induced by the user answer is not highly informative and does not reduce the region ${\mathcal{W}_\varLambda }$ significantly. Min-max based methods such as SMMR and MMD may be achieving a better performance level because they aim to computing queries that are informative whatever the user answer is.

It has been proven that the SMMR method generates the most informative query (Viappiani and Boutilier 2011) with respect to ${\mathcal{W}_\varLambda }$ if we consider all of the optimal solutions associated with ${\mathcal{W}_\varLambda }$. For each iteration of our framework, we considered only the solutions associated with the extreme points $\textit{Ext}({\mathcal{W}_\varLambda })$ of ${\mathcal{W}_\varLambda }$. The query computed by SMMR is the most informative only with respect tothe user preferences $\textit{Ext}({\mathcal{W}_\varLambda })$. We therefore cannot guarantee the optimality of the whole sequence of queries since different greedy methods (such as MMD) might generate a different set of extreme points from which we might extract more informative queries.

With MMD, we evaluated the minimum worst-case loss of a pair of solutions $s_{u}$ and $s_v$, composing a query only on the corresponding extreme points $u,v\in {\mathcal{W}_\varLambda }$. On the other hand, with SMMR, we evaluated the worst-case loss of the query rather than the same of the single solutions composing the query, and with respect to the whole set of extreme points $\textit{Ext}({\mathcal{W}_\varLambda })$. It is then interesting to see that in our experimental results, MMD was on average better in terms of the number of queries.

The presented computational results clearly show that the framework is very scalable with respect to the number of queries computed to achieve convergence. This measure grows fairly slowly with the size of the instance (see Table 4). This suggests the practical usability of the framework designed in the context of supplier selection.

**Table 4:** Experimental results

$|{\mathrm{I}}|$	$|{\mathrm{C}}|$	${{\rho }}$	$\alpha$ (%)	SMMR	MMD	MDS	SMMR	MMD	MDS
				$\mu _{time}$	$\mu _{time}$	$\mu _{time}$	$\mu _{query}$	$\mu _{query}$	$\mu _{query}$
10	30	0.2	100.0	1.12	**0**.**81**	0.96	**3**.**5**	4.0	4.0
10	30	0.3	100.0	3.79	**2**.**74**	2.84	**4**.**2**	4.4	4.4
10	30	0.4	100.0	19.28	8.19	**6**.**48**	7.2	7.0	**6**.**5**
10	30	0.5	100.0	37.72	**27**.**9**	36.41	**7**.**2**	8.0	10.5
10	40	0.2	100.0	0.97	**0**.**67**	0.85	**2**.**4**	**2**.**4**	3.1
10	40	0.3	100.0	7.12	**2**.**1**	2.45	4.9	**4**.**7**	5.1
10	40	0.4	100.0	15.13	**12**.**66**	17.52	**5**.**9**	6.4	7.8
10	40	0.5	100.0	42.31	**23**.**27**	35.59	7.7	**7**.**5**	8.9
10	50	0.2	100.0	3.98	**1**.**54**	2.36	**4**.**2**	4.9	5.3
10	50	0.3	100.0	3.77	**2**.**51**	2.56	**3**.**7**	3.8	4.0
10	50	0.4	100.0	24.86	17.83	**16**.**79**	**6**.**9**	7.0	7.1
10	50	0.5	100.0	127.23	85.34	**63**.**99**	11.1	10.6	**10**.**1**
10	60	0.2	100.0	2.9	**2**.**02**	2.64	**4**.**1**	4.2	4.4
10	60	0.3	100.0	2.21	**1**.**89**	2.04	**3**.**0**	3.2	4.0
10	60	0.4	100.0	59.37	**26**.**79**	28.15	7.8	**7**.**3**	8.0
10	60	0.5	100.0	62.45	57.57	**52**.**67**	**8**.**0**	8.4	8.6
20	30	0.2	100.0	87.09	**14**.**28**	17.9	10.0	**7**.**7**	9.1
20	30	0.3	100.0	118.3	**77**.**04**	96.23	10.4	**9**.**5**	12.1
20	30	0.4	100.0	305.89	**177**.**45**	200.36	11.8	**10**.**8**	11.5
20	30	0.5	100.0	350.1	372.71	**291**.**88**	11.4	11.1	**10**.**6**
20	40	0.2	100.0	51.09	18.59	**17**.**92**	8.3	**7**.**0**	**7**.**0**
20	40	0.3	100.0	204.49	132.89	**122**.**73**	10.3	**9**.**5**	10.0
20	40	0.4	100.0	305.93	**222**.**69**	370.6	11.4	**10**.**1**	12.3
20	40	0.5	100.0	672.57	**249**.**04**	313.46	10.8	**9**.**3**	12.1
20	50	0.2	100.0	62.95	23.35	**20**.**63**	8.4	7.3	**6**.**4**
20	50	0.3	100.0	326.81	171.18	**162**.**94**	11.4	**10**.**5**	11.0
20	50	0.4	100.0	297.36	**168**.**03**	325.06	10.3	**9**.**6**	11.1
20	50	0.5	100.0	**486**.**29**	571.8	552.89	**9**.**8**	11.7	12.7
20	60	0.2	90.0	21.79	**11**.**62**	21.1	**5**.**22**	**5**.**22**	5.89
20	60	0.3	100.0	171.13	122.38	**93**.**18**	**9**.**5**	10.2	10.1
20	60	0.4	100.0	**601**.**68**	642.37	742.42	**9**.**6**	10.9	11.4
20	60	0.5	90.0	574.19	**413**.**71**	1012.93	10.56	**9**.**89**	12.78
30	30	0.2	100.0	220.25	**39**.**43**	245.72	9.9	**7**.**1**	9.4
30	30	0.3	100.0	783.39	**289**.**54**	332.52	11.9	**10**.**9**	12.2
30	30	0.4	100.0	1390.38	405.88	**393**.**69**	11.9	**10**.**0**	11.3
30	30	0.5	100.0	469.37	329.37	**252**.**18**	**9**.**7**	10.0	10.2
30	40	0.2	100.0	663.05	345.26	**144**.**93**	11.1	**10**.**5**	10.6
30	40	0.3	100.0	**372**.**97**	505.3	621.16	**9**.**6**	10.9	12.3
30	40	0.4	80.0	1279.45	**827**.**41**	879.14	14.25	**12**.**5**	13.62
30	40	0.5	50.0	1236.04	1017.44	**711**.**69**	9.6	**9**.**4**	10.2
30	50	0.2	100.0	471.24	**167**.**48**	211.95	11.7	**9**.**5**	10.6
30	50	0.3	100.0	1301.86	1565.44	**609**.**45**	13.1	14.1	**12**.**5**
30	50	0.4	90.0	701.8	893.49	**321**.**11**	**9**.**22**	9.44	10.22
30	50	0.5	100.0	1086.3	1349.17	**948**.**75**	**10**.**5**	10.6	11.4
30	60	0.2	100.0	560.84	**302**.**85**	310.38	12.3	**10**.**6**	12.2
30	60	0.3	100.0	1383.75	**733**.**28**	991.63	11.9	11.2	**10**.**8**
30	60	0.4	80.0	1340.93	**550**.**73**	1418.09	11.12	**10**.**38**	13.25
30	60	0.5	70.0	1159.35	1130.19	**958**.**08**	11.71	**10**.**29**	13.57
Average values				2.079	**1**.**174**	1.34	1.076	**1**.**038**	1.144

The bold values represent the best result among the three methods in that row, with respect to the time (for the first set of three columns) and the number of queries (for the second set of three columns)

## Discussion

This paper presents a general framework for guiding decision makers via a query generation mechanism in a multi-criteria supplier selection process inspired by a real-world scenario. We assumed a preference model based on a weighted sum utility function, with the criteria evaluating the alternatives being cost, lateness, lead time and reputation.

This work lies between two research areas: supplier selection, a relevant topic in OM, and preference learning, a research area belonging to AI. On the one hand, it provides an alternative perspective to the solution of supplier selection problems. On the other hand, it presents an interactive preference elicitation approach using novel query selection strategies. Briefly, our procedure can be summarized as follows:

1. We solved a MILP problem with different weights to find a set of alternative solutions for the DM.
2. We asked the DM to express a preference between two solutions selected using a query selection strategy.
3. We used the DM’s response to reduce the uncertainty concerning the DM’s preference.
4. If we found an alternative with a max regret lower than a certain threshold, we recommended it.

The computational experimentation assessed the performance of our framework using three preference elicitation strategies to generate the queries, where two of the three were novel. We compared our novel query selection strategies with a myopically optimal query selection strategy based on setwise max regret. This had a similar number of interactions with the DM but with a much lower computational time.

In Sect. 7.1, we discuss the implications of the proposed framework for DMs. Section 7.2 is related to the implications of theory of the novel query selection strategies for the purpose of preference elicitation. We conclude with Sect. 7.3 suggesting some extensions that may be considered for future research.

### Implications for managers and decision-makers

The main advantage of our framework is the low cognitive effort required by the DM with respect to the standard MCDM approaches adopted in the supplier selection literature. These approaches, including AHP and ANP, are based on complex interviews to precisely define the weights representing the DM’s preferences. This is where the DM has to know details about the approach itself. Our framework is much simpler from a DM’s point of view since it is based on a series of queries, each asking the DM to express a preference between two solutions. For example, it may be implemented along with a graphical user interface showing the alternative solutions composing the query for each iteration while highlighting the differences and similarities to ease the decision. Our experiments show that the average number of queries that we need to achieve convergence is less than 15 in all of the groups of instances considered. This means that 15 binary queries replace complex interviews, achieving a considerable speeding up of the process and much less cognitive effort. On the other hand, our preference elicitation method assumes the orrect answers with respect to the preference model representing the DM’s preferences. This is a potential weak point of our framework, since a wrong answer could exclude the weighted vector corresponding to the DM’s real preferences, hence the corresponding optimal solution.

Although we tackled a specific problem, our framework can be applied to other optimization problems based on the user preferences. In fact, the preference elicitation module is independent of the specific problem that we have to solve. The supplier selection problem presented in Sect. 3 can be replaced by any other optimization problem so long as the objective function is a weighted sum of a fixed number of criteria, and the weighted vector represents the user preferences with respect to these criteria. Some examples of the domains of application include chemical process engineering (Rangaiah et al. 2020), flow shop scheduling (Murata et al. 1996), inventory control (Tsai and Chen 2017) and maintenance planning (Allah Bukhsh et al. 2019).

### Implications for theory

Other methods for query selection are based on a geometric view of the polytope representing the possible DM’s preferences where the intention is to generate queries that equally divide the polytope. To be effective, these methods require a similar scale among the criteria evaluating the alternative solutions, thus it is common to normalize the utility function. However, as we discussed in Sect. 5.1, it is not at all clear how one should normalize with our formulation of the problem, making the methods difficult to apply in our context.

In our framework, we adopted the max regret as a measure to evaluate alternative solutions with uncertainty regarding the DM’s preferences. A related measure, used for query selection, is the setwise max regret that evaluates the max regret of a set of solutions. In particular, the query set with a minimum setwise regret is a myopically optimal query with respect to the max regret criterion. This method is less sensitive to the change in scale since it evaluates the maximum potential loss of the DM’s utility function and thus it is not based on geometric considerations regarding the polytope representing the possible DM’s preferences. However, this method is computationally expensive since we would need to evaluate the setwise max regret of all possible query sets. For this reason, we have presented two novel query selection strategies, MMD and MDS, based on a novel measure that we call discrepancy. Intuitively, this measure evaluates the loss of a solution with respect to a specific weighted vector and a corresponding optimal solution. The idea is to compute a set of solutions corresponding to a discrete set of weighted vectors (the extreme points of the polytope representing the possible DM’s preferences in our specific case), and to select two solutions that are maximally different, i.e., that maximizes the mutual discrepancies with respect to the corresponding associate weighted vectors. From our experimental results, it seems that MMD performs better on average than the setwise minimax regret in terms of execution time. Furthermore, we also got a lower average number of queries to achieve convergence. MMD seems to be a good alternative to the setwise max regret, especially when the computational time to generate a query significantly affects the overall execution time.

### Future research directions

Some of the recent developments in MCDM the purpose of supplier selection are related to introducing fuzzy theory in order to manage data incompleteness/uncertainty with respect to the DM’s response. This feature is not currently included in the proposed framework. However, the possibility of allowing fuzzy answers to the queries may be an interesting future research direction. Furthermore, the type of queries included in the framework may be extended to allow the user to express preferences among a certain set of solutions. In this case, the queries are less intuitive but could lead to a reduction in the overall number of queries required.

Future research may also involve an extension of the combinatorial problem to a stochastic case where aspects like the stochastic demands of the components are included in the problem definition. This can be easily achieved by replacing the MILP model considered in Sect. 5.1 with a stochastic extension. In that case, the resulting model would be much more complex and it would take a longer time to solve the problem optimally.

It would be interesting to extend our framework in a multi-agent context where the purpose is to find a common solution between two conflicting agents. In this case, the weighted vector corresponding to an optimal recommendation needs to consider the tradeoffs of the different DMs, which may be conflicting.

Finally, another future research direction is related to adapting the framework to a case where the combinatorial problem is solved using heuristic algorithms with no optimality or quality guarantee. If we increase the size of the instances considered, the current MILP model would not scale well, and the high computing times would make the interaction with the DM impractical.

## Data Availability

All of the data used for the experimental testing was randomly generated. The code used for the random data generation was developed by us (see “Appendices A”, “B” and “C”).

## Random catalogue generation

This appendix describes how to generate a suppliers’ catalogue by assigning to each supplier a certain set of components, such that an overall density ${{\rho }}$ was enforced. This is in addition to a minimum number of components ${\lambda }_{j,min}=2$ being provided by each supplier. Each component is provided by at least one supplier.

The suppliers’ catalogue is represented by a $|{\mathrm{I}}| \times |{\mathrm{C}}|$ matrix ${\mathrm{\Psi }}$ where each element $({i},j)$ is equal to 1 if supplier ${i}$ can provide component $j$, 0 otherwise. As previously indicated in Sect. 5.1, ${\mathrm{C}}_{i}$ is the set of components supplied by supplier ${i}$. Similarly, let ${\mathrm{I}}_j$ be the set of suppliers providing component $j$. The following is the procedure used to randomly generate the matrix ${\mathrm{\Psi }}$:

1. Set each element $({i}, j)$ of ${\mathrm{\Psi }}$ to 0
2. For each supplier ${i}$ in ${\mathrm{I}}$ choose a random component $j$ in ${\mathrm{C}}$, add $j$ to ${\mathrm{C}}_{i}$, add ${i}$ to ${\mathrm{I}}_j$ and set the $({i}, j)$-th element of ${\mathrm{\Psi }}$ to 1.
3. For each component $j$ in ${\mathrm{C}}$ such that $|{\mathrm{I}}_j|=0$ choose two different random suppliers ${i}$ and ${i}'$, add $j$ to ${\mathrm{C}}_{i}$ and ${\mathrm{C}}_{{i}'}$, add $\{{i},{i}'\}$ to ${\mathrm{I}}_j$, set the $({i}, j)$-th and the $({i}', j)$-th elements of ${\mathrm{\Psi }}$ to 1.
4. For each component $j$ in ${\mathrm{C}}$ such that $|{\mathrm{I}}_j| = 1$, let ${\mathrm{I}}_j=\{{i}'\}$, choose random supplier ${i}\ne {i}'$, add $j$ to ${\mathrm{C}}_{i}$, add ${i}$ to ${\mathrm{I}}_j$, and set the $({i},j)$-th element of ${\mathrm{\Psi }}$ to 1.
5. Let *K* be the value ${{\rho }}\cdot |{\mathrm{C}}| \cdot |{\mathrm{I}}|$ rounded to the nearest integer.
6. Let $\varDelta$ be the number of elements of ${\mathrm{\Psi }}$ equal to 1.
7. Let $$k=K-\varDelta $$.
8. While $k>0$, pick a random ${i}\in {\mathrm{I}}$ and $j\in {\mathrm{C}}$. If the $({i}, j)$-th component of ${\mathrm{\Psi }}$ is equal to 0, then set this element to 1 and decrease *k* by 1 unit.

## Random database generator

This section describes how to compute a random database of past orders. This is used in the framework to simulate the possibility of predicting the lead time ${l}_{{i},j,{t}}$ and lateness $\delta _{{i},j,{t}}$ parameters of the MILP model by means of real data. This is a function of the triple supplier ${i}$, component $j$ and tariff ${t}$. We assume that each entry of the database is a random order $o_k$ represented by a tuple $\langle {i}(o_k), j(o_k), {q}(o_k), {l}(o_k), {\delta }(o_k) \rangle$, meaning that the supplier ${i}(o_k)$ received an order of quantity ${q}(o_k)$ of component $j(o_k)$, and provided the components with lead time ${l}(o_k)$ and lateness of ${\delta }(o_k)$.

The number of orders generated for each component $j\in {\mathrm{C}}$ supplied by supplier ${i}\in {\mathrm{I}}$ is sampled from a discrete uniform distribution in the set $\{5, \ldots , 15\}$. The quantity of each order $o_k$ related to component $j(o_k)$ is the nearest integer of a value sampled from the Gaussian distribution (where negative and null values are discarded) whose parameters $\mu _q$ and $\sigma _q$ are shown in Table 5, depending on the category of $j(o_k)$.

**Table 5:** Gaussian distribution parameters used to sample the quantity of a component for an order with respect of the component categories

	Cheap	Average	Expensive
$\mu _q$	1000	200	30
$\sigma _q$	250	50	7.5

Five different values $RD_{i}$, $RV1_{i}$, $RV2_{i}$, $RV3_{i}$ and $RV4_{i}$ are assigned to each supplier in order to model its ability to deliver on time and to compute the delay and lateness of its orders. These values are computed as follows:

- $RD_{i}$ is sampled using a uniform distribution from the interval [0, 1);
- $RV1_{i}$ and $RV2_{i}$ are sampled using a discrete uniform distribution from the set $\{10, \ldots , 30\}$;
- $RV3_{i}$ and $RV4_{i}$ are sampled using a discrete uniform distribution from the set $\{1, \ldots , 10\}$.

The lead time ${l}(o_k)$ of an order $o_k$ assigned to a supplier ${i}(o_k)$ with quantity $q={q}(o_k)$ is then computed by summing up two values sampled form the following distributions:

- A discrete uniform distribution from the set $\{2, \ldots , 20\}$;
- A Gamma distribution with mean $RV1_{i}\cdot max(\log _{10}(10\cdot q),1)$ and a standard deviation $\sigma _{{l}(o_k)}=RV2_{i}\cdot max(\log _{10}(10\cdot q),1)$

and summing them.

The lateness ${\delta }(o_k)$ of a random order $o_k$ supplied by supplier ${i}(o_k)$ and of quantity $q={q}(o_k)$ is 0 if the random number sampled between 0 and 1 is less than $RD_{i}$. This models the case where the order is not late. Otherwise, ${\delta }(o_k)$ is computed as a sample of a Gamma distribution with a mean of $\mu _{{\delta }(o_k)}=RV3_{i}\cdot max(\log _{10}(10\cdot q),1)$ and a standard deviation of $\sigma _{{\delta }(o_k)}=RV4_{i}\cdot max(\log _{10}(10\cdot q),1)$. Please note that the term $max(\log _{10}(10\cdot q),1)$ is used in the computation of both ${l}(o_k)$ and ${\delta }(o_k)$ in order to increase the mean and standard deviation for orders with high quantities involved.

## Lead-time and lateness predictor

This appendix describes a predictor to compute the expected lead time ${l}_{{i},j,{t}}$ (Eq. 13) and expected delay ${\delta }_{{i},j,{t}}$ (Eq. 14) of a triple supplier ${i}$, component $j$ and quantity interval ${t}$ given a database of past orders. Let us first suppose that we have an *objective order* $o_0=\langle {i}(o_0), j(o_0), {q}(o_0),$ ${l}(o_0), {\delta }(o_0) \rangle$ where ${i}(o_0)$, $j(o_0)$ and ${q}(o_0)$ are known and we want to estimate ${l}(o_0)$ and ${\delta }(o_0)$. As in “Appendix B”, we indicate with $o_k=\langle {i}(o_k), j(o_k), {q}(o_k), {l}(o_k), {\delta }(o_k) \rangle$ a past order, i.e., the k-th order of a database. The idea is to compute ${l}(o_0)$ and ${\delta }(o_0)$ as a weighted average of the lead times and delays of past orders where each weight depends on the similarity of the corresponding past order $o_k$ with $o_0$.

Two types of similarity measures are considered:

- The similarity between the quantities defined as $Sim_{q}({q}(o_k), {q}(o_0))=\frac{\min ({q}(o_k), {q}(o_0))}{\max ({q}(o_k), {q}(o_0))}$;
- The similarity between the components $Sim_j(j(o_k), j(o_0))$, defined to be 1 if $j(o_k)=j(o_0)$, defined to be 0.5 if the category of $j(o_k)$ and $j(o_0)$ is the same, and $Sim_j(j(o_k), j(o_0))=0.1$, otherwise.

These two similarity measures are used to compute two *sub-weights* for each past order $o_k$:

$$\begin{aligned} { w^k_{q}=\frac{Sim_{q}({q}(o_k), {q}(o_0))}{ \sum ^n_{p=1} Sim_{q}({q}(o_p), {q}(o_0))}\text { and }w^k_j=\frac{Sim_j(j(o_k), j(o_0))}{\sum ^n_{p=1} Sim_j(j(o_p), j(o_0))}, } \end{aligned}$$

where $p\in [1,n]$ are the indexes of all the past orders stored in the database. After computing $w^k_{q}$ and $w^k_j$ for each past order $o_k$, the lead time ${l}(o_0)$ and the delay ${\delta }(o_0)$ of the objective order $o_0$ are estimated as ${l}(o_0)=\sum ^n_{k=1}(0.5 w^k_{q}+ 0.5 w^k_j) {l}(o_k)$ and ${\delta }(o_0)=\sum ^n_{k=1}(0.6 w^k_{q}+ 0.4 w^k_j) {\delta }(o_k)$, where ${l}(o_k)$ and ${\delta }(o_k)$ are delay and lead time of the past order $o_k$. The weight of $w^k_{q}$ in the formula used to compute ${\delta }(o_0)$ is set to 0.6 in order to give slightly more importance to past orders with similar quantities rather than past orders with similar components.

Note that ${l}_{{i},j,{t}}$ and ${\delta }_{{i},j,{t}}$ represent an expectation of lead time and delay given a specific quantity interval, while the method described computes an estimated lead time and delay given a specific quantity. We manage this issue by estimating the lead time and delay of two objective orders $o'_0$ and $o''_0$, where the quantities ${q}(o'_0)$ and ${q}(o''_0)$ are the lower and the upper bounds of the range of quantities defining the quantity interval ${t}$ (see Table 3 in Sect. 6.1). The values of ${l}_{{i},j,{t}}$ and ${\delta }_{{i},j,{t}}$ are then computed by averaging the values predicted for $o'_0$ and $o''_0$ as follows: ${l}_{{i},j,{t}}=({l}(o'_0)+{l}(o''_0))/2$ and ${\delta }_{{i},j,{t}}=({\delta }(o'_0)+{\delta }(o''_0))/2$. Since the upper bounds of the last quantity intervals in Table 3 are not defined, we consider these values to be 1500, 300 and 50 for the categories cheap, average and expensive respectively.

## Declarations

### Code availability

We implemented the software used for testing our methodologies using the programming language Python 3.7. The only license-based library we used was Cplex 12.9. If requested, we can provide the source code of our software.
